# Combined transcriptome and metabolome analysis of the resistance mechanism of quinoa seedlings to *Spodoptera exigua*

**DOI:** 10.3389/fpls.2022.931145

**Published:** 2022-07-28

**Authors:** Junna Liu, Li Li, Yongjiang Liu, Zhiyou Kong, Ping Zhang, Qianchao Wang, Shunhe Cheng, Peng Qin

**Affiliations:** ^1^College of Agronomy and Biotechnology, Yunnan Agricultural University, Kunming, China; ^2^College of Natural Resources and Environment, Baoshan University, Baoshan, China; ^3^Institute of Agricultural Sciences, Yangzhou, China

**Keywords:** quinoa seedling stage, metabolome, transcriptome, insect resistance mechanism, *Spodoptera exigua*

## Abstract

Quinoa has attracted considerable attention owing to its unique nutritional, economic, and medicinal values. The damage intensity of *Spodoptera exigua* at the seedling stage of quinoa fluctuates with the crop’s biological cycle and the environmental changes throughout the growing season. In this study, we used independently selected quinoa seedling resistant and susceptible cultivars to investigate the difference between insect resistance and insect susceptibility of quinoa at the seedling stage. Samples were collected when *Spodoptera exigua* 45 days after planting the seedlings, and broad targeted metabolomics studies were conducted using liquid chromatography-mass spectrophotometry combined with transcriptomic co-analysis. The metabolomic and genomic analyses of the insect-resistant and insect-susceptible quinoa groups revealed a total of 159 differential metabolites and were functionally annotated to 2334 differential genes involved in 128 pathways using the Kyoto Encyclopedia of Genes and Genomes analysis. In total, 14 metabolites and 22 genes were identified as key factors for the differential accumulation of insect-resistant metabolites in quinoa seedlings. Among them, gene-LOC110694254, gene-LOC110682669, and gene-LOC110732988 were positively correlated with choline. The expression of gene-LOC110729518 and gene-LOC110723164, which were notably higher in the resistant cultivars than in the susceptible cultivars, and the accumulations of the corresponding metabolites were also significantly higher in insect-resistant cultivars. These results elucidate the regulatory mechanism between insect resistance genes and metabolite accumulation in quinoa seedlings, and can provide a basis for the breeding and identification of new insect-resistant quinoa cultivars as well as for screening potential regulatory metabolites of quinoa insect-resistant target genes.

## Introduction

Quinoa (*Chenopodium quinoa* Willd.) is an annual dicotyledonous self-pollinating herb in the quinoa subfamily of Amaranthaceae. Originating in the Andes of South America, it has a planting history of more than 5000−7000 years and has a reputation of being a “super grain” (Bhargava et al., 2006; Vega-Gálvez et al., 2010; Hariadi et al., 2011; Adolf et al., 2012; Roman et al., 2020). Quinoa is highly adaptable, and has high resistance to cold, drought, saline-alkali, and barren conditions as well as plant pathogens. It is a C3 crop that prefers cold and high altitudes (Jacobsen, 2003). Quinoa has attracted considerable attention owing to its comprehensive nutritional value, high functional value, and strong ecological adaptability and stability. The Food and Agriculture Organization of the United Nations has identified it as a non-genetically modified nutritional food and the only single crop that can completely meet human nutritional requirements (FAO, 1985; Repo-Carrasco et al., 2003; Escuredo et al., 2014). Quinoa also has bacteriostatic properties and can be used for the treatment of inflammation, high blood pressure and as a source of antioxidants (Jin and Wei, 2011; Zhang et al., 2011; Karki et al., 2013; Dong et al., 2020; Guo et al., 2020). In recent years, the nutritional and functional value of quinoa has become apparent, and its planting area has been expanding.

The number and species of phytophagous insects have also increased, harming plants. That is to say that phytophagous insects themselves harm crops, they can also attract other insects to gnaw on large areas and even enter the soil, harming the inter-root area and causing crop yield reduction and economic losses (Campos et al., 2013). *S. exigua* is an omnivorous pest, which mainly endangers the new leaves and stems of the host crop. The plant leaves are eaten, leaving notches, holes, or networks, and sometimes only leaf veins. In serious cases, it can result in the death of the seedlings and affect crop quality and yield (Chen and Ruberson, 2008; Chen et al., 2010). Studies have shown that plants can rapidly recognize and use their own defense mechanisms in response to phytophagous insect infestation, by activating the expression of defense genes and activating resistance-inducing signaling defense pathways and prompting the production of relevant defense compounds, thus exhibiting insect resistance (Howe and Jander, 2008; Wu and Baldwin, 2010; Erb et al., 2012; Aljbory and Chen, 2018), but to the detriment of plant growth and yield, leading plants to make a trade-off between growth and resistance (Ode, 2006; Rowen and Kaplan, 2016). That is, when plants are attacked by diseases and insect pests, plants can activate a variety of signal pathways, such as the regulation of plant hormones, the increase of cytosolic Ca^2+^ concentration, the increase of cellular reactive oxygen species, etc., through pattern recognition receptors on their cell surfaces and specific elicitor or effector of insect saliva, thus regulating the activities of defense-related genes (Wu and Baldwin, 2010). At the same time the plant’s own metabolism is altered, and the most obvious secondary substance that changes is the content of phenolic toxic compounds; the accumulation of total phenols is positively correlated to the resistance of the variety, the higher the phenolic accumulation, the more resistant the variety (Singleton and Kratzer, 1969; Leszczyński et al., 1985; Lattanzio et al., 2009); also flavonoids and alkaloids have been shown to be closely related to plant resistance to insects (Pimenta et al., 2014). In the case of serious insect damage, some quinoa cultivars can avoid or reduce damage and have the ability to compensate after damage by phytophagous insect (Turlings and Tumlinson, 1992). In addition, in the study of crop response to stress, there are four main types related to plant resistance to stress: WRKY (Chen et al., 2012), AP2/erebp (Tang et al., 2011), MYB (Pitzschke, 2012), and bZIP (Hossain et al., 2010, Zhang et al., 2011), which can mediate crop response to various stresses by regulating plant hormone signal transduction, phenylpropane biosynthesis and other pathways. Relative gene expression is induced by transcription factors that are stimulated by external factors through protein interactions and signal transduction pathways (Liu et al., 2001). Further studies have found that many insects can interfere with or even adapt to the induced plant defense response (Kant et al., 2015), which leads to the production of defensive plant secondary metabolites. Therefore, it is important to study the defense mechanisms of plants and effectively examine and utilize defense substances.

Biological processes are complex and integrated, and data from a single omics cannot analyze the macroscopic developmental process of biological systems; multi-omics techniques are used to identify and analyze the interactions of single and multiple genes in metabolic pathways. Metabolomics is used to study the metabolic basis of phenotypic phenomena in organisms and to resolve metabolic pathways (Patti et al., 2012; Putri et al., 2013; Rai et al., 2017). The metabolome is the downstream result of gene expression, while the transcriptome is the medium of gene expression. Along with studying the transcriptome and metabolome, analyzing and identifying the positive and negative relationships between genes and metabolites can more directly reflect the changes of the material itself (Shen et al., 2016; Hirasawa et al., 2018). Therefore, the multi-omics technique provides a good visualization of the relationship between insect resistance metabolites and genes in quinoa seedlings.

At present, research on quinoa insect resistance at the seedling stage mainly focuses on the identification of insect resistant cultivar, and there is a lack of research on its mechanism, which limits the mining and utilization of the insect resistance genes of quinoa. Breeding and utilizing insect-resistant cultivar is the most fundamental, economical, and effective method with the least impact on the environment. In this study, the insect-resistant and susceptible cultivars of quinoa at the seedling stage were subjected to ultra-high performance liquid chromatography-tandem mass spectrometry to study quinoa metabolomics. We aimed to accurately and quantitatively identify the insect-resistant metabolites of quinoa at the seedling stage using a combination of metabolomic and transcriptomic analyses to examine differences in metabolites and the role of related genes between insect-resistant and insect-susceptible quinoa cultivars at the seedling stage and to provide a theoretical basis for further mining and utilization of insect-resistant genes of quinoa plants.

## Materials and methods

### Materials

In this experiment, eight quinoa cultivars independently selected by Yunnan Agricultural University were divided into two groups, four seedlings of insect-resistant quinoa cultivars (Dian Quinoa-52-3, Dian Quinoa-2019130, Dian Quinoa-QA13-8, and Dian Quinoa-Qinghai Black) and four insect-susceptible quinoa cultivars (Dian Quinoa-14-1-3, Dian Quinoa-93-2, Dian Quinoa-Yellow 3-4, and Dian Quinoa-Black Quinoa Purple), each cultivar had 3 biological repetitions, and each group of samples had 12 biological repetitions, a total of 24 samples, for the experimental samples. Which were planted in a greenhouse at the modern agricultural education base of Yunnan Agricultural University, Xundian County, Kunming City, Yunnan Province (E 102° 41′, N 25° 20′) in early June 2020. The plots were arranged in randomized groups with an area of 20 m^2^ (10 m × 2 m), sown at a depth of 2−3 cm, quinoa row spacing 80 cm, plant spacing 40cm, one plot was one replication for each cultivar, 60 plants per replication, 3 replications, 180 plants in total. Insect-resistant and insect-susceptible quinoa cultivars were planted at the same time interval to determine the resistance of the plants. Average temperature of greenhouse: 25.6°C; average humidity: 48%; sunshine duration: about 10 h; sowing depth: 2∼3 cm; substrate: soil texture was loamy, soil type was red soil, and ground strength was medium. The quinoa was managed with regular water management and under the same management conditions. Also according to the actual fertilizer requirements of quinoa, 15 t/hm^2^ of organic fertilizer and 0.75 t/hm^2^ of compound fertilizer [urea (containing N 46%); diammonium phosphate (containing P_2_O_5_ 46%): potassium sulfate (containing K_2_O 40%) = 1:1:0.2] were applied to the quinoa cultivars. Four insect-susceptible quinoa cultivars were observed daily for the presence of insect pests, and four seedlings of insect-resistant quinoa cultivars resistance was recorded.

**FIGURE 1:**
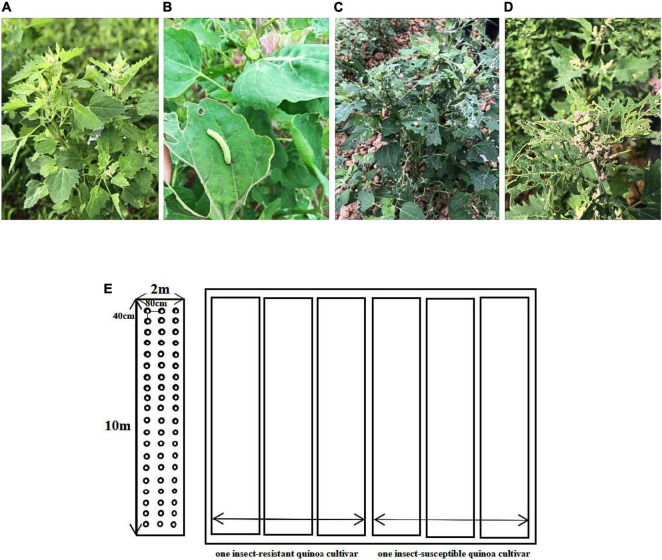
**(A)** Insect-resistant quinoa cultivars (R). **(B–D)** Insect-susceptible quinoa cultivars (N). **(E)** Map of cultivars planting plots.

Four insect-resistant cultivars (R) and four insect-susceptible cultivars (N) of quinoa at the seedling stage, when the emergence of *Spodoptera exigua* larvae became apparent in quinoa seedlings (45 days after planting), which were single marked on the same day and three independent plant leaves with the same growth stage were selected from each of the eight cultivars in R and N. In R, taking 10 g from each of the three different quinoa plants, for a total of 30 g of complete leaves from each cultivar. In N, taking 10 g from each of the three different quinoa plants, for a total of 30 g of *S. exigua* bitten leaves from each cultivar. The samples were immediately frozen in liquid nitrogen and stored at −80°C for transcriptome sequencing, metabolite determination and RT-qPCR analysis (Figures 1A–E).

### Metabolite extraction and detection

#### Sample preparation and metabolite extraction

The 24 samples (leaves) of insect-resistant and insect-susceptible quinoa seedling cultivars were freeze-dried in a vacuum and subsequently ground (30 Hz, 1.5 min) to powder using a grinder (MM 400, Retsch) respectively. Subsequently, 100 mg of powdered leaves was dissolved in 1.2 mL of 70% methanol extract and vortexed six times every 30 min for 30 s, respectively. After mixing separately, the samples were placed in a 4°C refrigerator overnight. After centrifugation at 12,000 rpm for 10 min, the supernatant was recovered, and the 24 samples were filtered respectively through a microporous membrane (0.22 μm pore size), and the supernatant were stored in an injection bottle for analysis via ultra-high performance liquid chromatography-mass spectrometry.

#### Qualitative and quantitative analysis of metabolites

The sample preparation, extraction analysis, and qualitative and quantitative analysis of metabolites of insect-resistant and insect-susceptible quinoa cultivars were performed according to the procedures described (Wang et al., 2019) of Wuhan Metware Biotechnology Co., Ltd.^1^ Based on the self-built database MWDB (metware database), Ultra Performance Liquid Chromatography, UPLC (SHIMADZU Nexera X2^2^) and Tandem mass spectrometry, MS/MS (Applied Biosystems 4500 QTRAP^3^) can accurately analyze the metabolites of samples qualitatively and quantitatively (Supplementary Material 1 and Supplementary Table 1). Quality control (QC) samples were prepared by mixing sample extracts. The repeatability of metabolite extraction and detection was judged by analyzing repeatability of samples under the same treatment. The results show high curve overlap of total ion flow for metabolite detection, i.e., consistent retention time and peak intensity, indicating good signal stability of mass spectrometry for the same sample detected at different times. The high stability of the instrument provides an important guarantee for the reproducibility and reliability of the data (TIC plots) (Supplementary Figure 1). Multivariate statistical analysis can be used to “simplify and downscale” the high-dimensional and complex data on the basis of the maximum retention of the original information, and to establish a reliable mathematical model to generalize and summarize the metabolic profile characteristics of quinoa (Eriksson et al., 2006). Principal component analysis (PCA) of the samples was conducted to preliminarily grasp the overall metabolic difference between the samples in each group and the degree of variation between the samples per group. The metabolite content data were normalized via unit variance scaling. Through correlation analysis between samples, the biological duplication between samples in the group was observed. The higher the correlation coefficient among samples in the group and between groups, the more reliable the differential metabolites are. We thus analyzed the metabolome data according to the orthogonal partial least squares discriminant analysis (OPLS-DA) model (Supplementary Figure 1) and drew the score and arrangement diagrams and further revealed the differences between each group (Thévenot et al., 2015). The Significantly different metabolites were screened based on VIP ≥1, fold change ≥2, and fold change ≤0.5 among the groups for further analysis. The identified differential metabolites were annotated through the Kyoto Encyclopedia of Genes and Genomes (KEGG) (Kanehisa and Goto, 2000) database.^4^ The significance was determined using the *p*-value of the hypergeometric test.

### Transcriptome sequencing and data analysis

#### RNA extraction, quantification, sequencing, and data analysis

Transcriptome sequencing, RNA extraction, RNA detection, library construction, online sequencing (Supplementary Material 2), and bioinformatics analysis were conducted by the Beijing Novogene Technology, Co., Ltd. (^5^ CN), which was further optimized based on the previous research and description (Zhu et al., 2018). Total RNA was extracted from insect-resistant and insect-susceptible cultivars of quinoa at the seedling stage. After RNA quality inspection and the library construction were completed, the sequencing was performed only after the test results met the requirements using Qubit 2.0 for preliminary quantification. Agilent 2100 was used to detect the insert size of the library. After the library was qualified, different libraries were pooled according to the target amount of the offline data and sequenced on the Illumina HiSeq platform (double terminal sequencing, and the length of each read was 150 bp). Using HISAT2, Clean Reads were compared with the reference genome to obtain Mapped Data. Fragments per kilobase of transcript per million fragments mapped (FPKM) was used as an index to measure the level of transcription or gene expression. The FPKM value of gene expression level ranged from 10^–2^ to 10^4^, and the screening condition of differential genes was | log_2_fold change| ≥ 1, and FDR < 0.05. Subsequently, DESeq2 (Robinson et al., 2010) was used to complete the analysis of differentially expressed genes, after which the total number of differentially expressed genes, the number of upregulated genes, and downregulated genes in each group were counted. In addition, hierarchical cluster analysis was performed, clustering heat map of each differential group was drawn, genes in the KEGG (Kanehisa et al., 2002, 2004) database were annotated, and the number of differential genes contained in each KEGG pathway was counted. Pathway significant enrichment analysis was performed using pathways in the KEGG database as units and applying hypergeometric tests to identify pathways that were significantly enriched in differentially expressed genes compared to the whole genomic background.

#### Real-time fluorescence quantitative PCR validation

To verify the reliability of the transcriptome sequencing results, all samples of all samples of genes with high expression on pathways related to insect resistance (three biological replicates) were selected for RT-qPCR experiments. The TUB-6 gene was selected as the internal reference gene, and the primers for the related genes used for RT-qPCR analysis were designed in Beacon Designer7.9. The PerfectStart SYBR qPCR Supermix (TransGen Biotech, Beijing, China) was used for RT-qPCR according to the manufacturer’s instructions. The reaction volume was 20 μL, including 2 × Perfectstarttm SYBR qPCR Supermix 10 μL, calibration solution 0.4 μL, nuclease free water (RNase free water) 6.8 μL, forward primer and reverse primer each primer (10 mm) 0.4 μL, cDNA 2 μL (200 μg/μL). The thermal cycle was as follows: the thermal cycle was as follows: 94°C (30 s), 94°C (5 s), 60°C (30 s), 40 cycles. And the relative gene expression level was calculated using the 2^–ΔΔCT^ method (Livak and Schmittgen, 2001). The specific methods were as follows: The CT value of the validated gene (C_T,Target_) and the CT value of the internal reference gene (C_T,TUB–6_) were calculated:


$$\Delta \,\text{CT}={\text{C}}_{\text{T},\text{Target}}-{\text{C}}_{\text{T},\text{TUB}-6},\,\Delta \,\Delta \,\text{CT}={\left({\text{C}}_{\text{T},\text{Target}}-{\text{C}}_{\text{T},\text{TUB}-6}\right)}_{\mathrm{Time}\,x}-\,{\left({\text{C}}_{\text{T},\text{Target}}-{\text{C}}_{\text{T},\text{TUB}-6}\right)}_{\text{Time}0.}$$

Note: Time x was any time point and Time 0 represents the 1 × expression of the target gene normalized to TUB-6. The mean CT values for both the target and internal control genes were determined at time zero.

We used SPSS 22.0 software for statistical analysis of differences between RT-qPCR and RNA sequencing results, with *p*-Values less than 0.05 being considered statistically significant and GraphPad Prism 8.0 software for visualization.

### New transcripts analysis

Cufflinks (v2.1.1) (Trapnell et al., 2010) can assemble the results of Tophat2 (v2.1.0) comparisons, and using Cuffcompare and known gene sequences, new unannotated genes or new exons of known genes can be identified, and the start and stop positions of known genes can also be optimized.

### Combined transcriptome and metabolome analysis

Principal component analysis of transcriptome and metabolome was performed to visualize whether differences existed between sample groups. Based on the results of differential metabolite and transcriptome differential gene analysis, differential genes and differential metabolites of the same grouping were mapped to the KEGG pathway map simultaneously to enable a better understanding of the relationship between genes and metabolites. Bar graphs were also plotted to show the enrichment level of the pathways with both differential metabolites and differential genes. Further correlation analysis of genes and metabolites detected in each differential grouping was performed, and Pearson correlation coefficients of genes and metabolites were calculated using the corr package in R. Significant differences in metabolites of genes with Pearson correlation coefficients greater than 0.8 in each difference grouping are shown by nine-quadrant plots. The O2PLS (Bouhaddani et al., 2016) model was used to integrate the analysis between the two data sets, reflecting the overall impact between different data sets, and additionally reflecting the weights of different variables in the model, so as to more precisely identify key regulatory phenomena. And multivariate statistical analysis method that reflects the overall correlation between the two sets of indicators using the correlation between integrated pairs of variables was used to perform (canonical correlation analysis, CCA) (González et al., 2008) analysis of the differential genes and differential metabolites in each pathway. Multiple biofunctional analysis identifies potential metabolites and corresponding differentially expressed genes at the molecular and biochemical levels by interactively comparing metabolomics and transcriptomics data.

## Results

### Qualitative and quantitative analysis of metabolites related to insect resistance cultivars in quinoa seedlings

**FIGURE 2:**
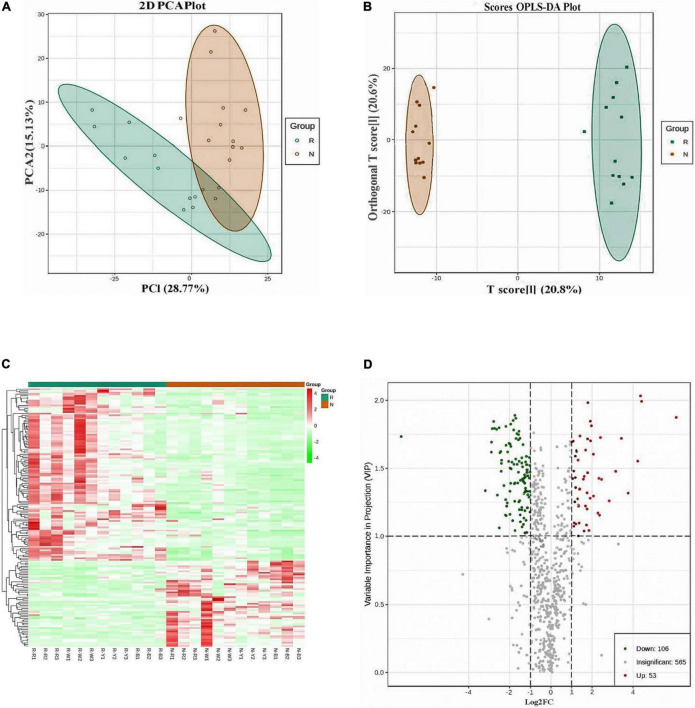
**(A)** Principal component analysis (PCA) score plot. **(B)** Orthogonal partial least squares discriminant analysis (OPLS-DA) score plot. **(C)** Heat map of the differential metabolite clustering. **(D)** Volcano plot of differential metabolites. The percentage of the PCA score plot indicates the explanation rate of this principal component to the data set. The horizontal and vertical coordinates in the OPLS-DA score plot show the gap between and within groups. The left side of the heat map of the differential metabolite clustering represents the differential metabolite clustering tree. The larger the abscissa and ordinate values in the volcano plot, the greater the difference of expression multiples between the two samples, and the more reliable the differentially expressed metabolites are.

Quantitative analysis of samples from R and N. A total of 724 metabolites were detected and quantified for the analysis of samples from R and N. The total ion flow plots (TIC plots) of the mass spectrometry detection analysis of different QC samples (Supplementary Figure 2) were analyzed via overlap display, and the results showed that the overlap of the curves of the total ion flow for metabolite detection was high, i.e., the retention time and peak intensity were consistent, indicating the high reliability of metabolite extraction and detection and good reproducibility of the data. From PCA score map (Figure 2A) and cluster analysis heat map (Figure 2C), the biological repeatability within the sample group was good, and the related metabolites between the groups had significant differences between the insect-resistant and susceptible cultivars of quinoa at the seedling stage. The correlation analysis between samples also showed good repeatability within the sample group (Supplementary Figure 3). The qualitative and quantitative detection of metabolites related to insect-resistant and insect-susceptible quinoa cultivars showed that 24 samples were selected for this project and divided into two groups for metabolomic studies, each group having 12 biological replicates. A total of 724 metabolites were detected, including 132 lipids, 121 phenolic acids, 119 flavonoids, 76 organic acids, 51 nucleotides and their derivatives, 80 amino acids and their derivatives, 47 alkaloids, 19 lignans and coumarin terpenoids, and 7 tannins (Table 1).

**TABLE 1:** Statistical classification of the number of differentially accumulated metabolites and differential genes.

Group	Number of differentially accumulated metabolites	Down regulated differentially accumulated metabolites	Up regulated differentially accumulated metabolites	Number of DEGs	Up regulated DEGs	Down regulated DEGs
R and N	159	53	106	2,334	758	1,576

In R, the number of differentially accumulated metabolites and DEGs were displayed. DEGs indicates differentially expressed genes.

### Analysis of relevant metabolite differences between insect-resistant and insect-susceptible quinoa cultivars at the seedling stage

Before the differential analysis, PCA (Figure 2A) and OPLS-DA (Figure 2B) were first performed on the grouped samples of the difference comparison. The distribution of each point shows that the separation trend between the groups was obvious, there were differences between the sample groups, and the sample repeatability within the group was good. From the OPLS-DA verification diagram, Q^2^ = 0.837 and the *p*-values of the models built in the group were less than 0.05, indicating that the prediction ability of the model was excellent, and that the model was the best fit. Based on the OPLS-DA results, the metabolites with different resistance or difference between groups can be preliminarily screened from the obtained variable importance in projection (VIP) of the OPLS-DA model for multivariate analysis. It can also be combined with the *p*-value or fold change of univariate analysis to further screen the differential metabolites. The combination of the fold change and VIP value of the OPLS-DA model was thus adopted to screen for differentially accumulated metabolites. Significantly different metabolites with fold change ≥2 and fold change ≤0.5 were selected. The differential metabolite clustering heat map (Figure 2C) clearly shows metabolite differences between insect-resistant and susceptible quinoa seedling cultivars. Each point in the differential metabolite volcano plot in N (Figure 2D) represents a metabolite, and a total of 724 metabolites were detected in R and N. A total of 159 (106 upregulated and 53 downregulated in R) metabolites were significantly different (Supplementary Table 2). We speculate that these 159 differential metabolites are main influencing metabolites between insect resistant and insect susceptible cultivars at seedling stage.

**FIGURE 3:**
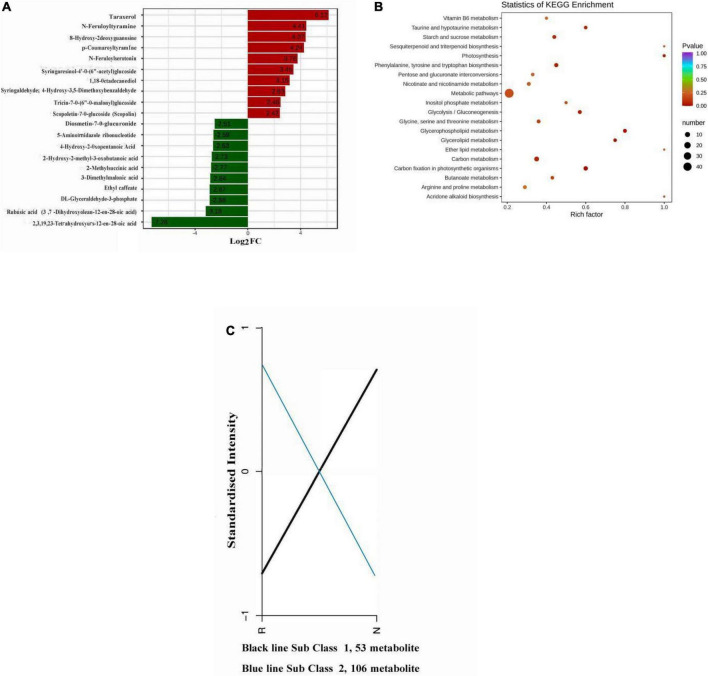
**(A)** Histogram of differential metabolite abundance. **(B)** Differential metabolite Kyoto Encyclopedia of Genes and Genomes (KEGG) enrichment graph. **(C)** Differential metabolite k-means graph. Log2FC and differential metabolites in the abscissa and ordinate in the histogram of differential metabolite abundance are shown. The color bar in **(B)** shows the range of *p*-value. The ordinate in the differential metabolite k-means graph represents the normalized relative content of metabolites.

Bar plot representing the differential expression analysis and KEGG enrichment graph of differential metabolites in each group (Figures 3A,B). Upregulation of the metabolite with the largest |log2FC| value among the differentially accumulated metabolites, there was a significant accumulation of metabolite differences between the two groups, and most of the metabolites in the top row of changes were alkaloids, phenolic acids and flavonoids; and downregulated differentially accumulated metabolites of organic acids, phenolic acids and terpenoids (Table 1). The metabolites with the largest |log2FC| values among the differentially accumulated metabolites were taraxerol, N-feruloyltyramine, p-coumaroyltyramine, scopoletin-7-o-glucoside (scopolin), 4-hydroxy-2-oxopentanoic acid and hydroxy-2-methyl-3-oxobutanoic acid (Figure 3A).

The average value of the relative content of differential metabolites in each group was standardized by z-score (Supplementary Figure 4), and the differential metabolites in different samples were normalized, and k-means clustering analysis was carried out. The k-means diagram of differential metabolites divided differential metabolites into two categories, and the changing trend of the relative content of standardized metabolites was obvious (Figure 3C). In sub class 2, 106 metabolites, R accounted for a larger proportion than N, we speculated that R contains some metabolites that make it insect-resistant, alkaloids, lipids, organic acids, phenolic acids, flavonoids, amino acids and their derivatives occupy a larger proportion. Typical stress-induced metabolic pathways were present, such as biosynthesis of amino acids, biosynthesis of secondary metabolites, phenylalanine, tyrosine and tryptophan biosynthesis, phenylpropanoid biosynthesis, glycospholipid metabolism, carbon fixation in photosynthetic organisms and plant hormone signal transmission (Figure 3B). Of the 106 constitutively upregulated metabolites in R (Figure 3C), only 41 differentially accumulated metabolites were annotated into the pathway. Among them, anthranilic acid was enriched in phenylalanine, tyrosine and tryptophan biosynthesis; caffeic acid and ferulic acid were enriched in phenylpropanoid biosynthesis; choline, o-phosphorylethanolamine and choline alfoscerate were enriched in glycospholipid metabolism; indole 3-acetic acid (IAA) was enriched in plant hormone signal transmission; dihydroxyacetone phosphate enriched in glycospholipid metabolism; 3-phospho-D-glyceric acid was enriched in biosynthesis of amino acids and carbon fixation in photosynthetic organisms; d-erythrose-4-phosphate was enriched in biosynthesis of amino acids (Supplementary Tables 3, 4). It was worth noting that methyl ferulate, ethyl caffeate, these 2 metabolites were not annotated into the pathway, but they were also important for insect resistance. These metabolites were accumulated in R compared to N. Among these, flavonoids and alkaloids are closely related to insect resistance, while amino acids and their derivatives are important precursors in the metabolic pathways that confer insect resistance.

### Transcriptome analysis of insect-resistant quinoa cultivars at the seedling stage

**FIGURE 4:**
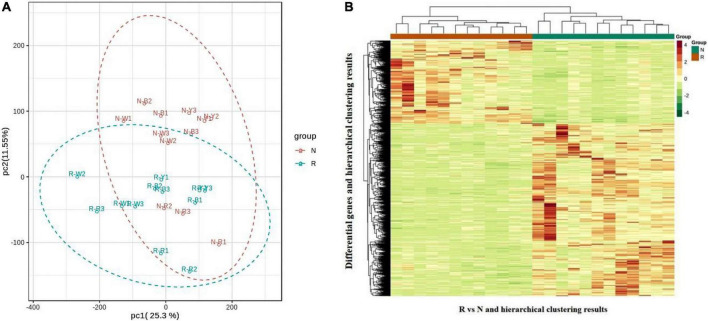
**(A)** Gene principal component analysis (PCA) map. **(B)** Differential gene clustering heat map. The abscissa and ordinate of differential gene clustering heat map represent the sample name, differential gene, and hierarchical clustering results.

The transcriptional analysis of Quinoa seedlings in R and N were divided into two groups for transcriptional studies after raw data filtering, sequencing error rate checking, and GC content distribution checking. A total of 156.02 Gb filtered sequencing data were obtained, with each sample reaching 6 Gb filtered sequencing data. The percentage of Q20 bases was 97% and above; the percentage of Q30 bases was 92% and above, and the GC content was higher than 43.0%. The proportion of sequenced reads that successfully matched the genome was higher than 70%, and the matching efficiency was higher than 90%. The efficiency of comparison between transcriptome data and reference genome was high (higher than 70%), which indicated that the reference genome was well assembled and that the transcriptome data measured in quinoa leaves were consistent with the reference genome, also indicating that the sequencing results were accurate and could be analyzed in the subsequent steps. The PCA plot (Figure 4A) and correlation heat map (Supplementary Figure 5A) of the three replicates of each group of samples were clustered together, indicating good stability of the method and high data quality. Pearson’s correlation coefficient r was used as the evaluation index of the repeated biological correlation. The closer the |r| value is to 1, the stronger the correlation between the two repeated samples. In this experiment, |r| was greater than 0.8 between biological replicate samples in both R and N. A clear separation was observed between the samples of the R and N. The quinoa samples of the R and N were biologically reproducible and distinct. The expression density distribution map (Supplementary Figure 5B) showed the trend and amounts of gene abundance in R and N, reflecting that the FPKM value of gene expression level is between log10^–2^ to log10^4^. The expression of FPKM was extracted after the centralization and standardization of differential genes; subsequently, hierarchical cluster analysis was performed, and a cluster heat map of each differential group (Figure 4B) was drawn to check the gene expression differences between R and N. The results showed that the differential genes between R and N were distinguished, indicating that our sequencing data were highly reliable.

### Analysis of transcriptome differences of insect-resistant quinoa seedling cultivars

**FIGURE 5:**
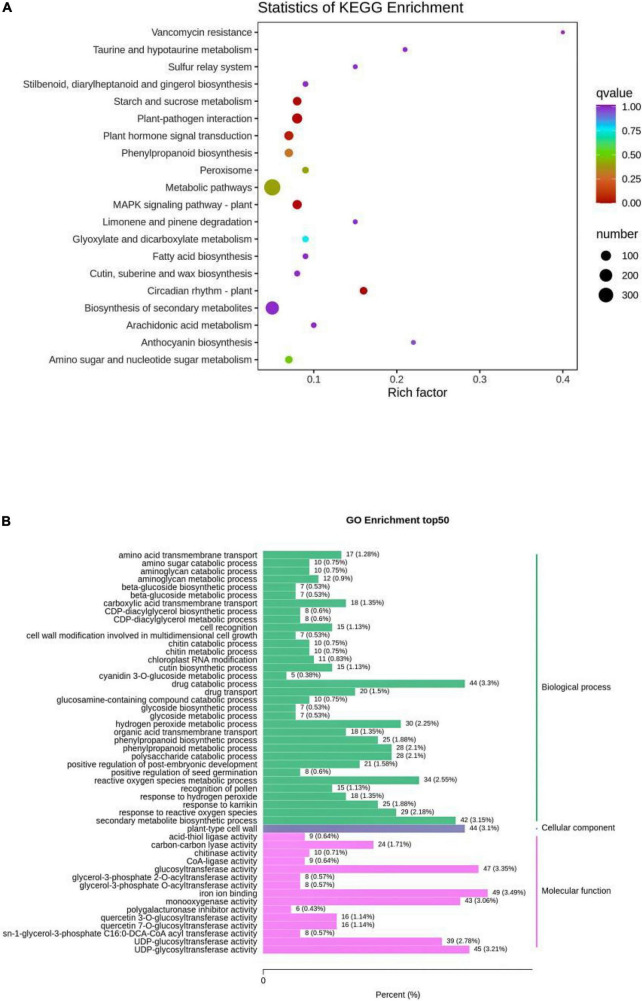
**(A)** Enrichment scatter plot. **(B)** Differential gene GO enrichment bar graph. The vertical coordinate represents the KEGG pathway. The horizontal coordinate indicates the rich factor, the larger the rich factor, the greater the enrichment. The larger the dot, the greater the number of differential genes enriched in the pathway. The redder the color of the dot, the more significant the enrichment. The abscissa of the column diagram of differential gene GO enrichment bar graph represents the proportion of the genes in the total number of genes annotated, and the ordinate represents the name of the go entry.

The detected genes were annotated in KEGG, Gene Ontology (GO), Non-Redundant Protein Sequence Database (NR), Swiss-Prot, KOG, Pfam, Tremble, and other databases. The results showed that KEGG was functionally annotated to 38,122 genes; GO to 38,862 genes; NR to 49,088 genes; Swiss-Prot to 32,844 genes; KOG to 46,755 genes; Pfam to 42,765 genes; and Tremble to 47,789 genes, involving 128 pathways. The analysis of differentially expressed genes was completed using DESeq2, and the differential gene filtering conditions were |log2Fold Change| ≥ 1 and FDR < 0.05. The total number of differential genes in R and N were 2,334, with 1,576 downregulated genes and 758 upregulated genes in R (Table 1 and Supplementary Table 5). Volcano plot to visualize the overall distribution of differential genes in the two sets of samples (Supplementary Figure 6). KEGG and GO enrichment analyses of differential genes were performed to help further understand the genetic differences in R and N. The degree of KEGG enrichment was measured using the rich factor, q-value, and the number of genes enriched to this pathway. Typical stress-induced significantly enriched pathway were present, such as starch and sucrose metabolism, plant hormone signal transduction, phenylpropanoid biosynthesis, MAPK signaling pathway plant and flavone and flavonol biosynthesis (Figure 5A). After screening differential genes, enrichment analysis was conducted to study the distribution of differential genes in Gene Ontology to clarify the embodiment of sample differences in gene function in the experiment. In GO enrichment analysis of differentially expressed genes, the 50 GO-Term with the lowest *q*-Value in the enrichment analysis results were selected and the bar graph of enrichment entries was plotted, and it was found that biological process 43%, cellular component 3.1%, molecular function 24.01% in R and N (Figure 5B), and 24, 16, 10 functional categories respectively (Supplementary Table 6). Through analysis, it was found that differentially expressed genes were enriched in the biological process of insect resistance in quinoa seedlings, indicating that the biological process played an important role in the mechanism of insect resistance. Further discovery in these significantly enriched pathways, the metabolites closely related to quinoa seedling insect resistance and the related genes were key genes to regulate insect resistance. Gene-LOC110722068 and IAA, gene-LOC110727583 and ferulic acid, and gene-LOC110704808 and choline had important effects on the expression of insect metabolites.

**FIGURE 6:**
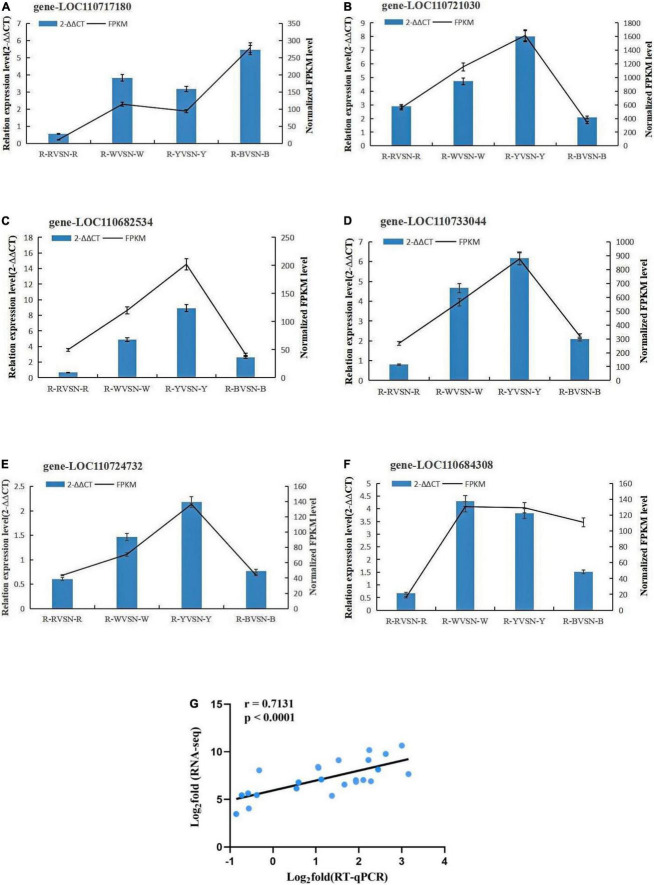
**(A–F)** Validation of the transcription levels for selected DEGs via RT-qPCR. **(G)** Verification of the expression patterns of RNA-seq results using RT-qPCR.

Genes with high expression in pathways related to insect resistance were selected for real-time fluorescence quantitative PCR with three replicates of each reaction, and 2^–ΔΔCT^ was used to analyze the normalized expression of each sample. In this way, we can calculate the 2^–ΔΔCT^ and SD, and at the same time calculate the FPKM and SD of the validated genes. Based on the 2^–ΔΔCT^ of the validated genes and the FPKM of the sequenced genes, the results showed that the expression trends detected by RT-qPCR were in good agreement with the RNA-seq data, which proved the reliability of the transcriptome data in this study (Figures 6A–G and Table 2).

**TABLE 2:** Primer sequences to validate genes.

Quantity	gene-ID	NCBI-gene ID	Primer	5′ to 3′
1	gene-LOC110682534	110682534	Forward Primer	GTTCCTTCCAGTTCTATC
			Reverse Primer	CTCTCCATCCTTATGTATC
2	gene-LOC110684308	110684308	Forward Primer	CGATTGTGTATGGAGAATA
			Reverse Primer	GGCTTGCTTAGTTGATTA
3	gene-LOC110717180	110717180	Forward Primer	GAGACAACTTACCATCAC
			Reverse Primer	GAACCTACCAACTGAATG
4	gene-LOC110721030	110721030	Forward Primer	CTATTCACTTCCACCATC
			Reverse Primer	TTAGCCTCCATAACATTC
5	gene-LOC110724732	110724732	Forward Primer	TCATCTCAGGAAGAACAT
			Reverse Primer	TTATTCGCATCAGAAGGA
6	gene-LOC110733044	110733044	Forward Primer	AGTATGAGTGTTTCTATGAG
			Reverse Primer	CTCTTCTCCACATTATCC
Internal reference gene	TUB-6	831100	Forward Primer	TGAGAACGCAGATGAGTGTATG
			Reverse Primer	GAAACGAAGACAGCAAGTGACA

### Analysis of insect resistance transcription factors in quinoa seedlings

Transcriptional genes were classified into 46 families, and the main transcription factors in this study included MYB, BHLH, WRKY, Hsf, and bZIP (Supplementary Table 7). Among the transcription factors, bZIP or gene-LOC110729518 was regulated by the plant G-box-binding factor and positively correlated with choline; gene-LOC110723164 was regulated by transcription factor HY5 and positively correlated with d-erythrose-4-phosphate (Table 3), indicating that these two genes played a key role in the regulation of metabolites in R and N at the quinoa seedling stage.

**TABLE 3:** Correlation analysis of differential metabolites and transcription factors.

Transcription factors	gene_ID	EC	Meta Name	Compounds	PCC	PCCP
bZIP	gene-LOC110729518	plant G-box-binding factor	pmb0484	Choline	0.841	2.638E−07
bZIP	gene-LOC110723164	transcription factor HY5	Zmzn000079	D-erythrose-4-phosphate	0.811	1.5092E−06

EC indicates enzyme digestion sites in related pathways, PCC indicates Pearson’s correlation coefficient.

### Analysis of insect resistance new transcripts in quinoa seedlings

In this study we also did a *de novo* gene analysis, using StringTie to assemble the reads into transcripts based on the location of the reads on the matched genomes. The spliced transcripts were then compared with the annotation information of the genome using GffCompare to identify new transcripts or new genes. In this study, 4368 new genes were identified (Supplementary Table 8).

### Combined transcriptome and metabolome analysis of insect resistance mechanisms in quinoa seedlings

**FIGURE 7:**
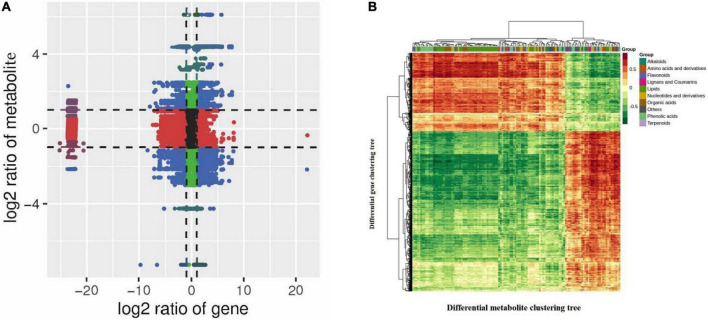
**(A)** Correlation analysis nine quadrant diagram. **(B)** Correlation coefficient clustering heat map. In the KEGG enrichment analysis, the *p*-Value histogram shows the enrichment degree of pathways with both differential metabolites and genes. In the correlation analysis, nine-quadrant plot analysis shows the difference multiple of gene metabolites with Pearson correlation coefficient greater than 0.8 in each difference group, which is divided into 9 quadrants from left to right and top to bottom, using black dashed lines. Each dot represents a gene/metabolite, black dots indicate non-differential metabolites and genes, blue dots indicate genes and metabolites that are both significantly different (up or downregulated), red dots indicate genes whose transcriptomes are significantly different but whose metabolomes are not, and green dots indicate metabolites whose metabolomes are significantly different but whose transcriptomes are not. For differential metabolites with correlation coefficient above 0.8, select all the correlation calculation results and draw the correlation coefficient cluster heat map.

To understand the differences in quinoa seedling synthesis in R and N, we integrated transcriptomic data and metabolomic data for analysis and mapped both differential genes and differential metabolites to the KEGG pathway map. The KEGG pathway map revealed the pathways closely associated with insect resistance and the significantly different associated genes and metabolites in R and N of insect-resistant quinoa cultivars. Gene and metabolite correlation clustering heat map has more than 0.8 correlation of differential metabolites and differential genes for mapping, it can be clearly seen that the gene and metabolite positive and negative correlation was strong. Quadrant 3, 7 refers to genes and metabolites with a consistent pattern of differential expression and a consistent trend of regulation, with changes in metabolites likely to be positively regulated by genes, such as indole 3-acetic acid (IAA), dihydroxyacetone phosphate, d-erythrose-4-phosphate, scopoletin-7-o-glucoside(scopolin), choline, o-phosphorylethanolamine, choline alfoscerate, anthranilic acid; quadrants 1, 2, 4 refer to genes and metabolites with higher metabolite accumulation than genes and non-consistent regulatory trends, where metabolites are upregulated and genes are unchanged or down-regulated, such as scopoletin-7-o-glucoside (scopolin); quadrants 6, 8, 9 refer to genes and metabolites with a non-consistent regulatory trend, where the gene is upregulated and the metabolite is unchanged or downregulated, such as glucose-1-phosphate, dihydroxyacetone phosphate, 3-phospho-D-glyceric acid, d-erythrose-4-phosphate, caffeic acid, ferulic acid, o-phosphorylethanolamine, choline alfoscerate (Figure 7A and Supplementary Tables 9–11). The associated genes and metabolites were mainly enriched in plant hormone signal transduction, starch and sucrose metabolism, carbon fixation in photosynthetic organisms, phenylpropanoid biosynthesis, glycerophospholipid metabolism, phenylalanine, and tyrosine and tryptophan biosynthesis, and there were 71, 57, 7, 54, 17, and 2 significantly different genes, respectively (Supplementary Figure 7A).

Among them, in the KEGG enrichment analysis, we could see the synthetic differences between the two groups of the insect-resistant and insect-susceptible cultivars by comparing the two groups of quinoa cultivars (Figure 7B and Table 4). In the plant hormone signal transduction pathway, indole-3-acetic acid (IAA) was significantly high accumulation in insect-resistant cultivars (R) of quinoa at seedling stage than insect-susceptible quinoa cultivars (N), and IAA was upregulated expression in R. Meanwhile, corresponding gene-LOC110695735 of IAA was upregulated expression in R. While the two corresponding genes of IAA, the expression of gene-LOC110725292 and gene-LOC110683095 were R lower than N, the two genes were downregulated expression in R. And by correlation analysis ribonuclease T2 [EC:3.1.27.1] (gene-LOC110695735) was positively correlated with IAA (PCC = 0.804), indicating that this gene positively affected the accumulation of IAA, which belongs to the indole alkaloids and plays an important role in insect-resistant quinoa cultivars. Brassinosteroid insensitive 1-associated receptor kinase 1 [EC:2.7.10.12.7.11.1] (gene-LOC110725292, BAK1) and 3-o-methyltransferase [EC:2.1.1.68] (gene-LOC110683095, COMT) were negatively correlated with IAA (PCC = −0.834, −0.83), indicating that these two genes affected the accumulation of IAA.

**TABLE 4:** Metabolite expression and its correlation.

Meta name	Compounds	R	N	log2FC	gene_ID	R	N	log2FC
pme1651	Indole 3-acetic acid (IAA)	23793.25	9408.71	−1.3385	gene-LOC110725292 gene-LOC110683095 gene-LOC110695735	0.73 6.77 54.26	2.22 29.57 23.49	1.5640 2.1300 −1.1980
mws1090	Glucose-1-phosphate	3138466.67	1536465.83	−1.0304	gene-LOC110682146 gene-LOC110737753	2.43 4.39	7.52 10.99	1.6830 1.3360
pme3313	D-fructose6-phosphate	558772.50	212063.58	−1.3978	gene-LOC110682146	2.43	7.52	1.6830
Zmzn000078	Dihydroxyacetone phosphate	119184.00	26406.86	−2.1742	gene-LOC110735748	2.99	14.55	2.2730
Zmgn000447	3-phospho-D-glyceric acid	780377.50	264345.67	−1.5617	gene-LOC110682146	2.43	7.52	1.6830
Zmzn000079	D-erythrose-4-phosphate	108879.92	52589.33	−1.0499	gene-LOC110721904 gene-LOC110699613	0.81 11.64	0.26 26.51	−1.6040 1.2150
mws2212	Caffeic acid	1044960.00	232799.83	−2.1663	gene-LOC110734008	0.37	2.66	2.7070
mws0014	Ferulic acid	4903901.67	1367665.00	−1.8422	gene-LOC110727583	1.84	7.63	2.0230
mws1077	Scopoletin-7-o-glucoside (scopolin)	33149.19	176816.17	2.4152	gene-LOC110718932 gene-LOC110735213 gene-LOC110724454	0.00 2.84 7.09	0.31 22.77 17.25	4.8300 3.0210 1.2860
pmb0484	Choline	18269975.00	8388466.67	−1.1230	gene-LOC110694254 gene-LOC110682669 gene-LOC110732988	52.56 3.20 5.79	10.57 0.55 0.95	−2.3260 −2.3530 −2.4800
mws0704	O-phosphorylethanolamine	45876.42	19604.53	−1.2266	gene-LOC110729416	19.00	8.64	−1.1080
mws0120	Choline alfoscerate	142287.50	32442.83	−2.1328	gene-LOC110725292	0.73	2.22	1.5640
mws1078	Anthranilic Acid	421410.00	185970.83	−1.1801	gene-LOC110729895 gene-LOC110725292	0.71 0.73	1.85 2.22	1.3380 1.5640
Lmbn002862	3-hydroxybenzoic acid	2806134.17	1372185.83	−1.0321	gene-LOC110729180	5.60	21.75	1.9960

Log2FC that is the logarithm base 2 of fold change (FC) of the differential metabolite; positive log2FC indicates up-regulation, while negative indicates down-regulation. R and N in metabolites indicate differentially accumulated metabolites; R and N in genes indicate differentially expressed genes.

In carbon fixation in photosynthetic organisms, the accumulation of d-erythrose-4-phosphate was R higher than N in Rvs.N, d-erythrose-4-phosphate was upregulated expression in R. The gene corresponding to d-erythrose-4-phosphate, the expression of gene-LOC110721904 was R higher than N, this gene was upregulated expression in R. While gene-LOC110699613 was R lower than N in Rvs.N, this gene was downregulated expression in R. Correlation analysis revealed that s-adenosylmethionine decarboxylase [EC:4.1.1.50] (gene-LOC110721904) was positively correlated with d-erythrose-4-phosphate (PCC = 0.828), indicating that expression of this gene promotes the formation of d-erythrose-4-phosphate. While galacturan 1,4-alpha-galacturonidase [EC:3.2.1.67] (gene-LOC110699613) was negatively correlated with d-erythrose-4-phosphate (PCC = −0.826), indicating that weak expression of this gene affects the accumulation of d-erythrose-4-phosphate.

The accumulation of 3-phospho-D-glyceric acid was R higher than N in Rvs.N, 3-phospho-D-glyceric acid was upregulated expression in R. The gene corresponding to 3-phospho-D-glyceric acid, the expression of gene-LOC110682146 was R lower than N, this gene was downregulated expression in R. Correlation analysis revealed that ATP-dependent RNAhelicaseDDX41 [EC:3.6.4.13] (gene-LOC110682146) was negatively correlated with 3-phospho-D-glyceric acid (PCC = –0.817), indicating that weak expression of this gene affects the accumulation of 3-phospho-D-glyceric acid.

In the phenylpropanoid biosynthesis pathway (Supplementary Figure 7B), the accumulation of caffeic acid was R higher than N in Rvs.N, caffeic acid was upregulated expression in R. The corresponding gene-LOC110734008 of caffeic acid was R lower than N, and this gene was downregulated expression in R. Correlation analysis revealed that thioredoxin reductase (NADPH) [EC:1.8.1.9] (gene- LOC110734008) was negatively correlated with caffeic acid (PCC = –0.815), indicating that weak expression of this gene affected the accumulation of caffeic acid. The accumulation of scopoletin-7-o-glucoside (scopolin) was R lower than N in Rvs.N and scopoletin-7-o-glucoside (scopolin) was downregulated expression in R. The expression of gene-LOC110718932, gene-LOC110735213 and gene-LOC110724454 were R lower than N and these three genes were downregulated expression in R. Correlation analysis revealed galactan beta-1,4-galactosyltransferase[EC:2.4.1.-] (gene-LOC110718932), inositol-hexakisphosphate/diphosphoinositol - pentakisphosphate 1-kinase [EC:2.7.4.24] (gene-LOC110735213) and maleylacetoacetate isomerase [EC:5.2.1.2] (gene-LOC110724454) with scopoletin-7-o-glucoside (scopolin) were positively correlated with caffeic acid (PCC = 0.826, 0.816, 0.81), indicating that weak expression of these three genes affected the accumulation of scopoletin-7-o-glucoside (scopolin). The accumulation of ferulic acid was R higher than N in Rvs.N, ferulic acid was upregulated expression in R. The expression of gene-LOC110727583 was R lower than N, this gene was downregulated expression in R. Correlation analysis revealed that ferredoxin-chelate reductase [EC:1.16.1.7] (gene-LOC110727583) was negatively correlated with ferulic acid (PCC = −0.8), indicating that weak expression of this gene affected the accumulation of ferulic acid.

In the glycerophospholipid metabolic pathway, the accumulation of choline was R higher than N in Rvs.N, and choline was upregulated expression in R. The expression of gene-LOC110694254, gene-LOC110682669 and gene-LOC110732988 were R higher than N, these three genes were upregulated expression in R. Correlation analysis revealed that beta-amylase [EC:3.2.1.2] (gene-LOC110694254), cytosolic prostaglandin-E synthase [EC:5.3.99.3] (gene-LOC110682669) and inositol 3-alpha-galactosyltransferase [EC:2.4.1.123] (gene-LOC110732988) were positively correlated with choline (PCC = 0.83, 0.819, 0.805), indicating that the upregulation of these three genes promotes choline accumulation. In phenylalanine, tyrosine and tryptophan biosynthetic pathways, the accumulation of anthranilic acid was R higher than N in Rvs.N, and anthranilic acid was upregulated expression in R. The expression of gene-LOC110729895 and gene-LOC110725292 were R lower than N, the two gene were downregulated in R. Correlation analysis revealed that phosphofructokinase 1 [EC:2.7.1.11] (gene-LOC110729895) and brassinosteroid insensitive 1-associated receptor kinase 1 [EC:2.7.10.1 2.7.11.1] (gene-LOC110725292) were negatively correlated with anthranilic acid (PCC = −0.832, −0.855), indicating that weak expression of these two genes affected the accumulation of anthranilic acid (Figure 7A and Table 5).

**TABLE 5:** Correlation analysis of differential metabolites and differential genes.

gene_ID	Meta Name	Compounds	EC	Enzyme	PCC
gene-LOC110683095	pme1651	Indole 3-acetic acid (IAA)	EC:2.1.1.68	caffeic acid 3-O-methyltransferase	−0.83
gene-LOC110725292	pme1651	Indole 3-acetic acid (IAA)	EC:2.7.10.1 2.7.11.1	brassinosteroid insensitive 1-associated receptor kinase 1	−0.834
gene-LOC110695735	pme1651	Indole 3-acetic acid (IAA)	EC:3.1.27.1	ribonuclease T2	0.804
gene-LOC110682146	mws1090 pme3313 Zmgn000447	Glucose-1-phosphate fructose6-phosphate 3-phospho-D-glyceric acid	EC:3.6.4.13	ATP-dependent RNA helicase DDX41	−0.821 −0.817 −0.817
gene-LOC110737753	mws1090	Glucose-1-phosphate	EC:2.7.10.1 2.7.11.1	brassinosteroid insensitive 1-associated receptor kinase 1	−0.805
gene-LOC110735748	Zmzn000078	Dihydroxyacetone phosphate	EC:1.1.1.195	cinnamyl-alcohol dehydrogenase	−0.817
gene-LOC110721904	Zmzn000079	D-erythrose-4-phosphate	EC:4.1.1.50	S-adenosylmethionine decarboxylase	0.828
gene-LOC110699613	Zmzn000079	D-erythrose-4-phosphate	EC:3.2.1.67	galacturan 1,4-alpha-galacturonidase	−0.826
gene-LOC110734008	mws2212	Caffeic acid	EC:1.8.1.9	thioredoxin reductase (NADPH)	−0.815
gene-LOC110727583	mws0014	Ferulic acid	EC:1.16.1.7	ferric-chelate reductase	−0.8
gene-LOC110718932	mws1077	Scopoletin-7-o-glucoside (scopolin)	EC:2.4.1.-	galactan beta-1,4-galactosyltransferase	0.826
gene-LOC110735213	mws1077	Scopoletin-7-o-glucoside (scopolin)	EC:2.7.4.24	inositol-hexakisphosphate/diphosphoinositol-pentakisphosphate 1-kinase	0.816
gene-LOC110724454	mws1077	Scopoletin-7-o-glucoside (Scopolin)	EC:5.2.1.2	maleylacetoacetate isomerase	0.81
gene-LOC110694254	pmb0484	Choline	EC:3.2.1.2	beta-amylase	0.83
gene-LOC110682669	pmb0484	Choline	EC:5.3.99.3	cytosolic prostaglandin-E synthase	0.819
gene-LOC110732988	pmb0484	Choline	EC:2.4.1.123	inositol 3-alpha-galactosyltransferase	0.806
gene-LOC110729416	mws0704	O-phosphorylethanolamine	EC:3.1.27.1	ribonuclease T2	0.801
gene-LOC110725292	Mws0120	Choline alfoscerate	EC:2.7.10.1 2.7.11.1	Brassinosteroid insensitive 1-associated receptor kinase 1	−0.82
gene-LOC110729895	mws1078	Anthranilic acid	EC:2.7.1.11	6-phosphofructokinase 1	−0.832
gene-LOC110729180	Zmzn000079	D-erythrose-4-phosphate	EC:1.6.2.2	cytochrome-b5 reductase	−0.802

EC indicates enzyme digestion sites in related pathways, PCC indicates Pearson’s correlation coefficient.

## Discussion

In this study, the metabolome and transcriptome were combined to resolve the differences between quinoa seedling resistance to *S. exigua*. Interactions between quinoa, phytophagous insect and environmental factors are complex and diverse. This study found that *S. exigua* was obvious pest in the quinoa seedling stage, which infests the plant with larvae that need to continuously nibble on quinoa leaves to ensure their own nutrition, but at the same time, the nibbled leaves of *S. exigua* and its saliva can cause quinoa produce secondary metabolites and that can affect growth. In quinoa-*S. exigua* intercropping, we observed that plants of the insect-resistant quinoa cultivars always maintained a healthy growth state from the seedling stage to the mature stage and were not disturbed by pests throughout the growth period. However, the insect-susceptible cultivars seedlings were attacked by *S. exigua*, which nibbled on their leaves and thus mutilated and affected seedling growth, while the pest continuously grew. Secondary metabolites produced by plants play a key role in plant adaptation to environmental stresses. Studies have shown that quinoa can produce secondary metabolites (SMs), such as phenolic acids, alkaloids and flavonoids. These metabolites are toxic and difficult for phytophagous insects to feed, in this case, they may have the potential to lead to lower damage of phytophagous insects (Kokanova-Nedialkova et al., 2009). While under the potential influence of phytophagous insects, the plant also produces secondary metabolites to defend itself against phytophagous insects, such as flavonoids, lignans and saponins (Kokanova-Nedialkova et al., 2009, Murphy and Matanguihan, 2015).

The quinoa seedling R and N were further determined using ultra performance liquid chromatography tandem mass spectrometry and a total of 724 metabolites were detected, with a total of 159 differential metabolites (Supplementary Table 1). Plant hormone signal transduction, starch and sucrose metabolism, carbon fixation in photosynthetic organisms, phenylpropanoid biosynthesis, glycerophospholipid metabolism, phenylalanine, tyrosine and tryptophan biosynthesis are closely related to insect resistance. Studies have found that phenolic acids and alkaloids are closely related to plant insect resistance and that amino acids and their derivatives are important precursors in the metabolic pathway of plant insect resistance (Eriksson et al., 2006; Goyal et al., 2012). The content of alkaloids and phenolic acids in the metabolites of insect-resistant cultivars were much higher than that of insect-susceptible cultivars. In addition, glycerophosphodiester phosphodiesterase regulates choline alfoscerate and choline accumulation and enrichment in glycerophospholipid metabolism; caffeic acid 3-o-methyltransferase was enriched in phenylpropane biosynthesis and regulated caffeic acid and ferulic acid accumulation, which further indicated significant differences between insect-resistant and insect-susceptible cultivars of quinoa at seedling stage. Indoles and ferulic acid, secondary metabolites produced by phenylpropane, which have very important roles in plant resistance to stress and pests and diseases, and phenolic acids are aromatic secondary metabolites synthesized in large quantities by plants, including ferulic acid and caffeic acid, all of which have strong defensive functions (Ti and Zhang, 2009; Murakami et al., 2014). It has been shown that ferulic acid analogs are closely related to the cell wall synthesis of plants, and feruloylation of the cell wall can fight pathogens and insects to some extent, and high concentrations of ferulic acid may inhibit aphid feeding, survival and reproduction and enhance plant resistance (Lyons et al., 1993; Cabrera et al., 1995). In other words, some secondary metabolites produced inside the plant such as Indole 3-acetic acid (IAA), choline alfoscerate, choline, anthranilic acid, d-erythrose-4-phosphate and caffeic acid make *S. exigua* difficult to feed, and the external plant refers to feruloyls with plant feruloylation of the cell wall to enhance its own resistance. Also phenolic ferulic acids lignify the cells, all of which can be achieved against pathogens and insects, meaning that quinoa was resistant to insects as other studies have similar conclusions (Cabrera et al., 1995; Eriksson et al., 2006; Goyal et al., 2012; Murakami et al., 2014).

While alkaloids have been shown to be a class of substances with insecticidal activity that can affect the insect nervous system, inhibit insect feeding and insect egg laying and thus have an impact on normal insect growth and development of insects (Pedras et al., 2002; Clauss et al., 2006). In the present study, choline was positively correlated with gene-LOC110694254 (PCC = 0.83), gene-LOC110682669 (PCC = 0.819), and gene-LOC110732988 (PCC = 0.806), and also expressed significantly higher in R than N, choline belongs to alkaloids closely related to insect resistance, which means that these three genes regulate the accumulation of choline to resist the bite of phytophagous insects. Secondly, in the immunization process of quinoa seedlings against biological stress transcription factors play an important role by regulating the expression of target genes. When the target genes are overexpressed in plants, they can transcribe and activate the expression of a series of downstream anti-insect functional genes. Genes do not respond to induction alone, but genes and metabolites in plants often intersect and influence each other, in which bZIP also plays an important role (Zhang et al., 2011). Gene-LOC110729518 in the transcription factor bZIP was positively correlated with choline (PCC = 0.841), and gene-LOC110723164 was also positively correlated with d-erythrose-4-phosphate (PCC = 0.811), The regulated metabolites of these two genes are closely related to insect resistance. In this study, to elucidate the differential regulation of insect-resistant bioaccumulation in different cultivars of quinoa at the seedling stage, The association analysis of differential metabolites and genes between Rvs.N showed that 14 species of differential metabolites and 22 differential genes were closely related to insect resistance, which was closely related to the expression level of key genes in the bioaccumulation of insect-resistant quinoa cultivars. However, the present study is about combined transcriptome and metabolome analysis of the resistance mechanism of quinoa seedlings (between insect-resistant and insect-susceptible cultivars) to *S. exigua.* In addition, quinoa comes in many colors (red, white, yellow and black quinoa) and different colors of quinoa have different insect resistance, but the research on the resistance difference between different colors of quinoa between insect-resistant and insect-susceptible cultivars have not been understood in depth. At the same time, 4368 novel genes were identified in this study (Supplementary Table 8), but further validation analyses are needed.

## Conclusion

We integrated transcriptomic and metabolomic analyses to elucidate the different regulatory pathways of biosynthesis between insect-resistant quinoa cultivars and insect-susceptible quinoa cultivars. 6 metabolic pathways were identified namely plant hormone signal transduction, starch and sucrose metabolism, carbon fixation in photosynthetic organisms, phenylpropanoid biosynthesis, glycerophospholipid metabolism, phenylalanine, and tyrosine and tryptophan biosynthesis.

A total of 159 differential metabolites were detected, and 2,334 differential genes annotated via the KEGG function, involving 128 pathways. The association analysis of differential metabolites and the transcriptome revealed 14 differential metabolites and 22 differential genes between R and N, among which, gene-LOC110694254, gene-LOC110682669, and gene-LOC110732988 were positively correlated with choline; the expression of gene-LOC110729518 and gene-LOC110723164, in relation to the bZIP transcription factor in insect-resistant quinoa cultivars, was significantly higher than that in susceptible cultivars, while the accumulation of corresponding metabolites in insect-resistant cultivars was also significantly higher than that in insect-susceptible cultivars. Thus, these may be the key factors responsible for the difference in the insect-resistant bioaccumulation in quinoa seedlings. Owing to the significant difference in the expression of key factors in insect-resistant biosynthesis of quinoa seedlings, the difference in metabolites such as alkaloids and phenolic acids may affect the formation of insect-resistant quinoa cultivars. The findings of this study will be helpful for breeders to select new insect-resistant quinoa cultivars.

## Data availability statement

The datasets presented in this study can be found in online repositories. The names of the repository/repositories and accession number(s) can be found below: https://www.ncbi.nlm.nih.gov/, PRJNA837037, SRP375030.

## Author contributions

JL wrote the original draft and performed the methodology. LL wrote the original draft and carried out the formal analysis. YL did the conceptualization, and wrote, reviewed, and edited the manuscript. ZK carried out the formal analysis, performed the methodology, and visualized the data. PZ collected the field samples and prepared the plant materials. QW carried out the formal analysis and investigated the data. SC and PQ supervised the data and carried out the project administration and funding acquisition. All authors contributed to the article and approved the submitted version.

## Footnotes

1
www.metware.cn

2
https://www.shimadzu.com.cn/

3
http://www.appliedbiosystems.com.cn/

4
http://www.kegg.jp/kegg/compound/

5
www.novogene.com

## Supplementary material

Supplementary Figure 1 Orthogonal partial least squares discriminant analysis model.

Supplementary Figure 2 Ion flow plots (TIC plots) of the mass spectrometry detection. **(A)** N for negative ion mode; **(B)** P for positive ion mode. This is the spectrum obtained by summing the intensities of all ions in the mass spectra at each time point and depicting them consecutively.

Supplementary Figure 3 Correlation diagram between samples. The abscissa represents the sample name, the ordinate represents the corresponding sample name, the color represents the correlation value, and the group is the group.

Supplementary Figure 4 *Z*-value diagram of differential metabolites. The horizontal coordinate is the value after normalization of the relative content of the substance, the vertical coordinate is the name of the metabolite, and the points of different colors represent different groups of samples.

Supplementary Figure 5 **(A)** Correlation heat map. **(B)** Expression density distribution. The square of correlation heat map| r| between biological repeated samples must at least be greater than 0.8. The curves of different colors in the Expression density distribution represent different samples, and the abscissa represents the logarithm of the corresponding sample FPKM.

Supplementary Figure 6 Differential gene expression volcano plot. The horizontal coordinates indicate changes in gene expression level and the vertical coordinates indicate the level of significance of differentially expressed genes. Red dots represent upregulated differential genes, green dots represent downregulated differential genes and blue dots represent non-differentially expressed genes.

Supplementary Figure 7 **(A)** Kyoto Encyclopedia of Genes and Genomes (KEGG) enrichment analysis, *p*-Value histogram. **(B)** Phenylpropanoid biosynthesis canonical correlation analysis (CCA) plot. In the KEGG enrichment analysis, the p-value histogram shows the enrichment degree of pathways with both differential metabolites and genes. Phenylpropanoid biosynthesis canonical correlation analysis (CCA) plot is divided into four regions. In the same region, the farther away from the origin, the closer the data points are to each other, and the higher the correlation.

## Abbreviations

BHLH: basic helix-loop-helix
bZIP: basic region-leucine zipper
DEG: differentially expressed gene
FDR: false discovery rate
FPKM: fragments per kilobase per million mapped reads
GO: Gene Ontology
KEGG: Kyoto Encyclopedia of Genes and Genomes
MYB: myeloblastosis
CCA: canonical correlation analysis
OPLS-DA: orthogonal partial least squares discriminant analysis
PCA: principal component analysis
QC: quality control
RT-qPCR: real time quantitative PCR
TF: transcription factor
TIC: total ion current
UPLC-MS/MS: ultraperformance liquid chromatography-tandem mass spectrometry
O2PLS: two-way orthogonal partial least squares.
