# Supplementary material for: Combined transcriptome and metabolome analysis of the resistance mechanism of quinoa seedlings to Spodoptera exigua
Source: Front Plant Sci. 2022 Jul 28;13:931145. doi: 10.3389/fpls.2022.931145 (PMC9370066; doi:10.3389/fpls.2022.931145)
Supplement: Supplementary file 1 [file Data_Sheet_13.PDF]

Chromatographic mass spectrometric acquisition conditions and experimental methods:

Provided by Wuhan Metway Biotechnology Co. Detection and based on the self-built database MWDB (metware database).
The data acquisition instrumentation system consists mainly of Ultra Performance Liquid Chromatography (UPLC) (SHIMADZU Nexera X2, https://www.shimadzu.com.cn/) and Tandem Mass Spectrometry (MS/MS) (Applied Biosystems 4500 QTRAP, https://www.shimadzu.com.cn/). spectrometry (MS/MS) (Applied Biosystems 4500 QTRAP, http://www.appliedbiosystems.com.cn/).

1. Sample preparation and extraction
Biological samples are freeze-dried by vacuum freeze-dryer (Scientz-100F). The freeze-dried sample was crushed using a mixer mill (MM 400, Retsch) with a zirconia bead for 1.5 min at 30 Hz. Dissolve 100mg of lyophilized powder with 1.2ml 70% methanol solution, vortex 30 seconds every 30 minutes for 6 times in total, place the sample in a refrigerator at 4°C overnight. Following centrifugation at 12000rpm for 10 min, the extracts were filtrated (SCAA-104, 0.22μm pore size; ANPEL,Shanghai, China, http://www.anpel.com.cn/) before UPLC-MS/MS analysis.

2. UPLC Conditions
The sample extracts were analyzed using an UPLC-ESI-MS/MS system (UPLC, SHIMADZU Nexera X2, www.shimadzu.com.cn/; MS, Applied Biosystems 4500 Q TRAP, www.appliedbiosystems.com.cn/). The analytical conditions were as follows, UPLC: column, Agilent SB-C18 (1.8 µm, 2.1 mm*100 mm); The mobile phase was consisted of solvent A, pure water with 0.1% formic acid, and solvent B, acetonitrile with 0.1% formic acid. Sample measurements were performed with a gradient program that employed the starting conditions of 95% A, 5 % B. Within 9min, a linear gradient to 5% A, 95% B was programmed, and a composition of 5% A, 95% B was kept for 1min. Subsequently, a composition of 95% A,5.0 % B was adjusted within 1.10 min and kept for 2.9 min. The flow velocity was set as 0.35ml per minute; The column oven was set to 40°C; The injection volume was 4 μL. The effluent was alternatively connected to an ESI-triple quadrupole-linear ion trap (QTRAP)-MS.

3. ESI-Q TRAP-MS/MS
LIT and triple quadrupole (QQQ) scans were acquired on a triple quadrupole-linear ion trap mass spectrometer (Q TRAP), AB4500 Q TRAP UPLC/MS/MS System, equipped with an ESI Turbo Ion-Spray interface, operating in positive and negative ion mode and controlled by Analyst 1.6.3 software (AB Sciex). The ESI source operation parameters were as follows: ion source, turbo spray; source temperature 550°C; ion spray voltage (IS) 5500 V (positive ion mode)/-4500 V (negative ion mode); ion source gas I (GSI), gas II(GSII), curtain gas (CUR) were set at 50, 60, and 25.0 psi, respectively; the collision-activated dissociation(CAD) was high. Instrument tuning and mass calibration were performed with 10 and 100 μmol/L polypropylene

glycol solutions in QQQ and LIT modes, respectively. QQQ scans were acquired as MRM experiments with collision gas (nitrogen) set to medium. DP and CE for individual MRM transitions was done with further DP and CE optimization. A specific set of MRM transitions were monitored for each period according to the metabolites eluted within this period.

Software for processing de LC/MS data:

Analysis	Software	Version
PCA	R(base package)	3.5.0
Pearson Correlation Coefficients	R(base package)	3.5.0
Heatmap	R(pheatmap)	1.0.12
OPLS-DA	R(MetaboAnalystR)	1.0.1

Information on the processing parameters used by the software：

1. PCA

Unsupervised PCA (principal component analysis) was performed by statistics function prcomp within R (www.r-project.org). The data was unit variance scaled before unsupervised PCA.

2. Hierarchical Cluster Analysis and Pearson Correlation Coefficients

The HCA (hierarchical cluster analysis) results of samples and metabolites were presented as heatmaps with dendrograms, while pearson correlation coefficients (PCC) between samples were caculated by the cor function in R and presented as only heatmaps. Both HCA and PCC were carried out by R package pheatmap. For HCA, normalized signal intensities of metabolites (unit variance scaling) are visualized as a color spectrum.

3. Differential metabolites selected

Significantly regulated metabolites between groups were determined by VIP >= 1 and absolute Log_2FC (fold change) >= 1. VIP values were extracted from OPLS-DA result, which also contain score plots and permutation plots, was generated using R package MetaboAnalystR. The data was log transform (log_2) and mean centering before OPLS-DA. In order to avoid overfitting, a permutation test (200 permutations) was performed.

4. KEGG annotation and enrichment analysis

Identified metabolites were annotated using KEGG Compound database (http://www.kegg.jp/kegg/compound/), annotated metabolites were then mapped to KEGG Pathway database (http://www.kegg.jp/kegg/pathway.html). Pathways with significantly regulated metabolites mapped to were then fed into MSEA (metabolite

sets enrichment analysis), their significance was determined by hypergeometric test's p-values.
