# Supplementary figures and images for: Combined transcriptome and metabolome analysis of the resistance mechanism of quinoa seedlings to Spodoptera exigua
Source: Front Plant Sci. 2022 Jul 28;13:931145. doi: 10.3389/fpls.2022.931145 (PMC9370066; doi:10.3389/fpls.2022.931145)

**Supplementary Figure 1**

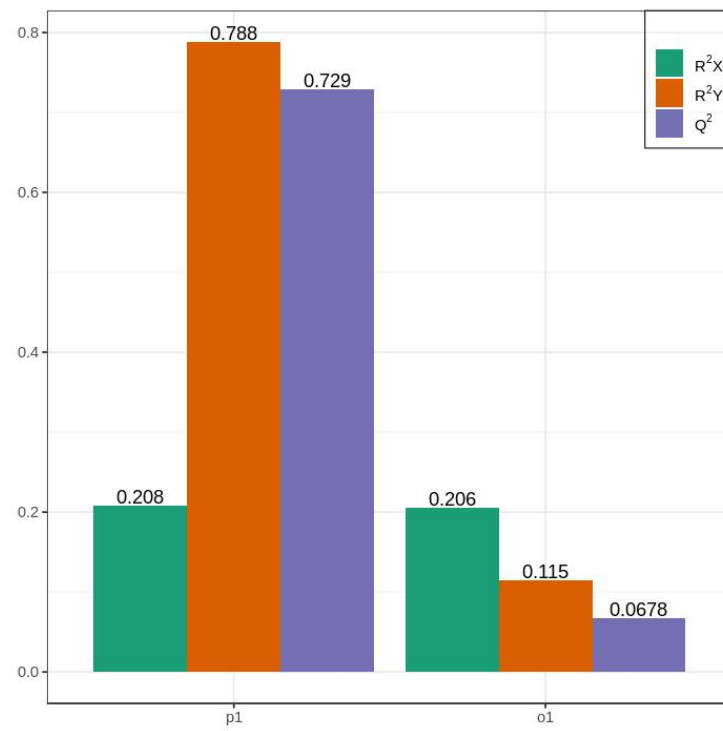

Supplement: Supplementary Figure 1 — Orthogonal partial least squares discriminant analysis model. [file Data_Sheet_6.PDF]

Supplementary Figure 2

(A)

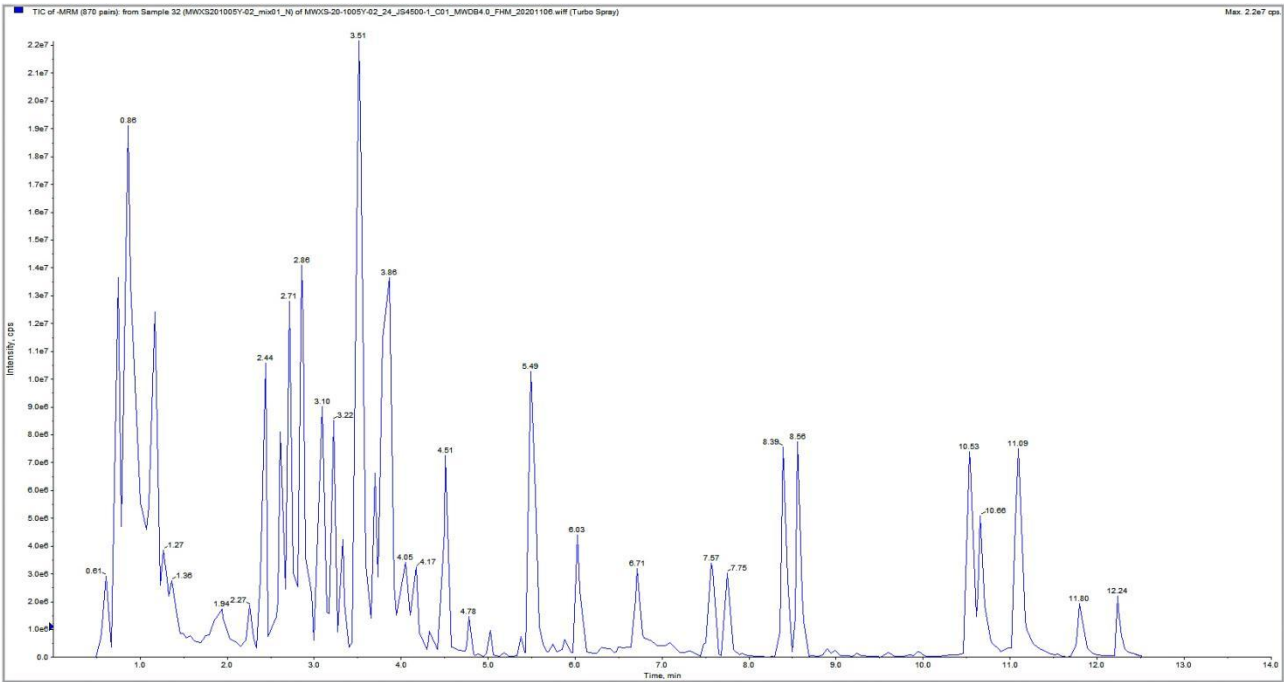

(B)

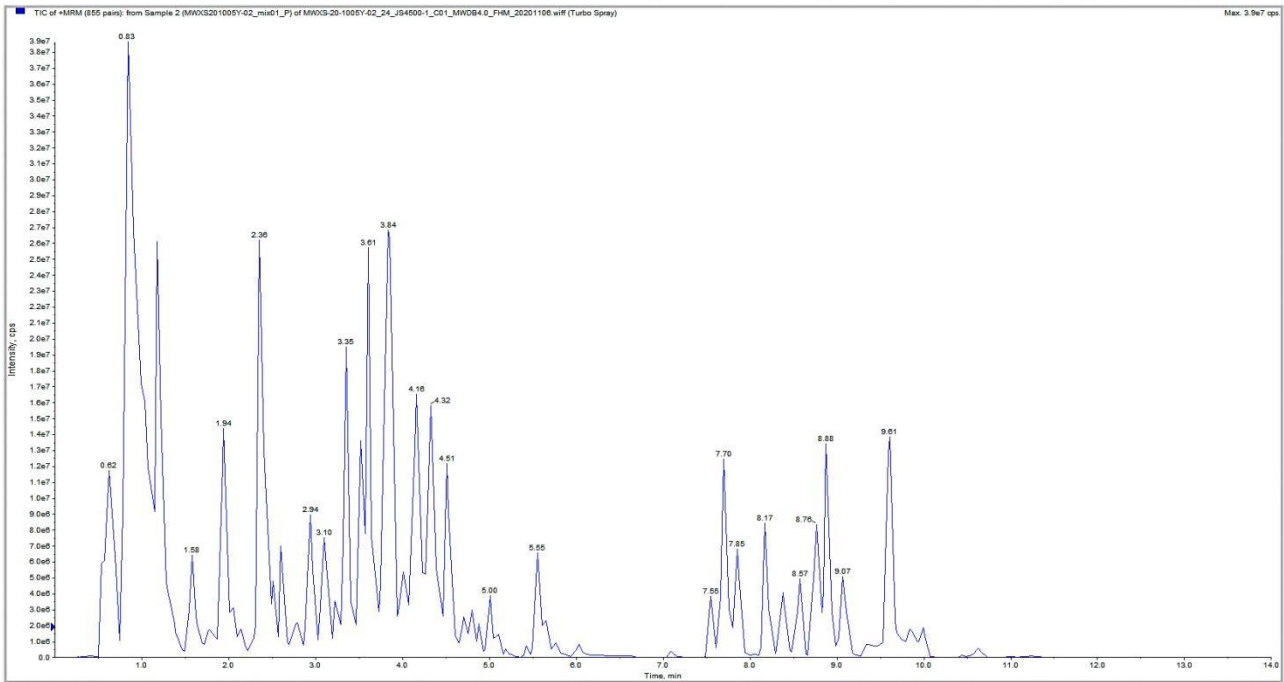

Supplement: Supplementary Figure 2 — Ion flow plots (TIC plots) of the mass spectrometry detection. (A) N for negative ion mode; (B) P for positive ion mode. This is the spectrum obtained by summing the intensities of all ions in the mass spectra at each time point and depicting them consecutively. [file Data_Sheet_7.PDF]

Supplementary Figure 3

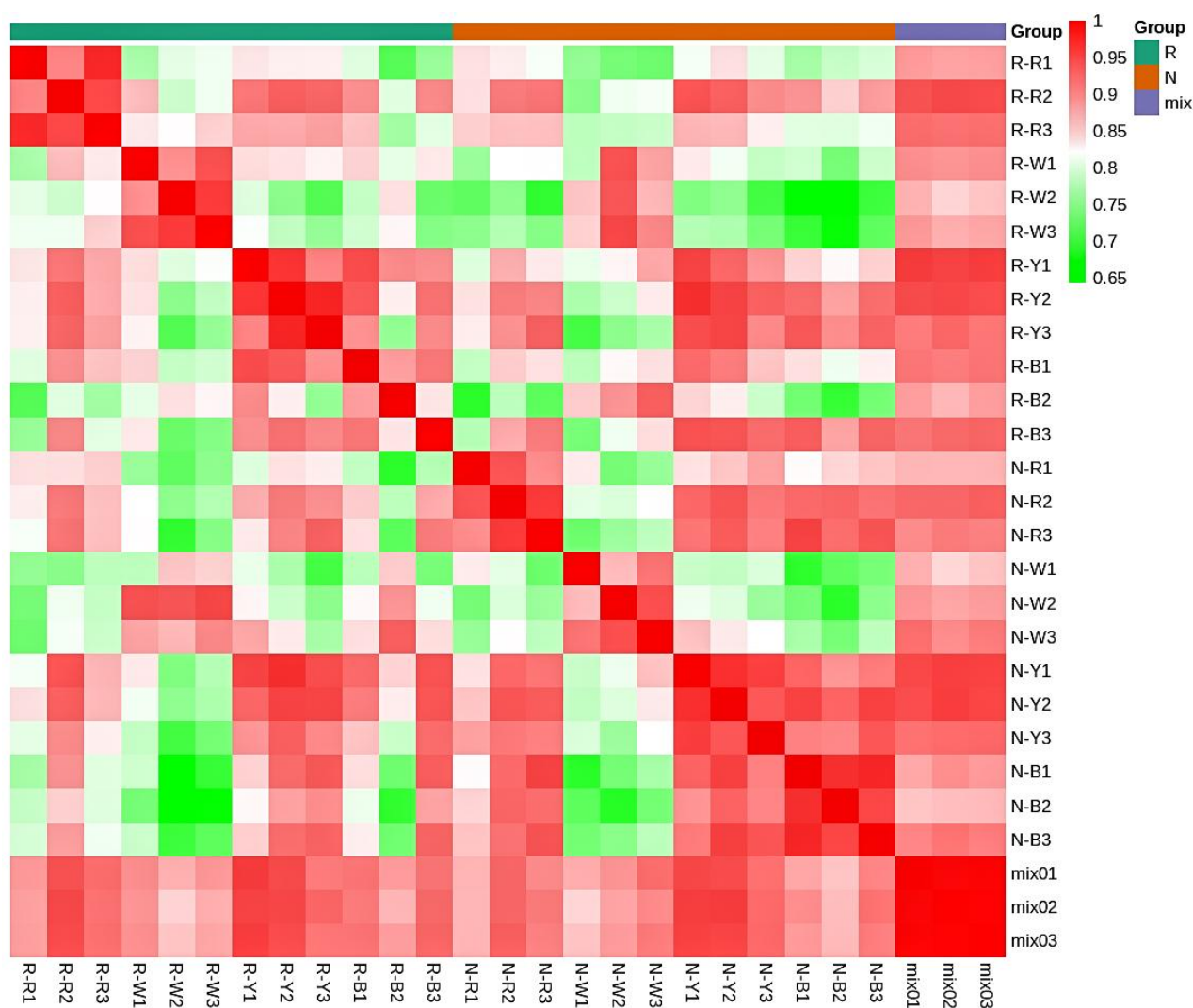

Supplementary Figure 4

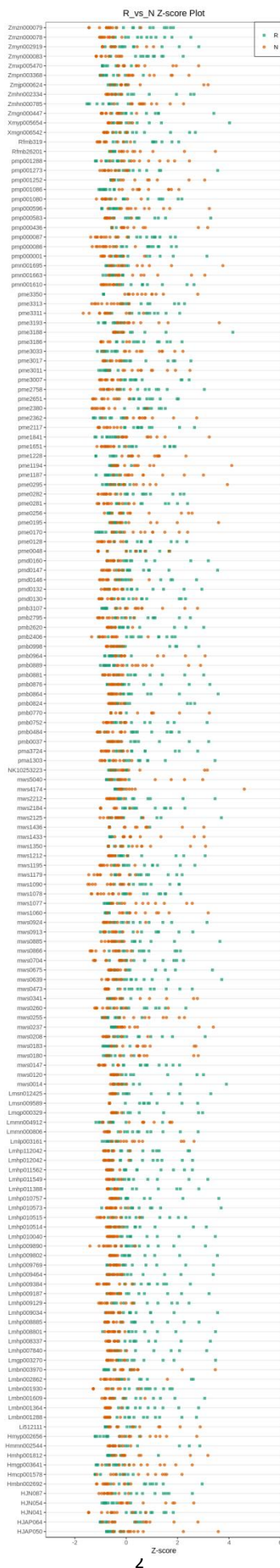

2

Supplementary Figure 5

(A)

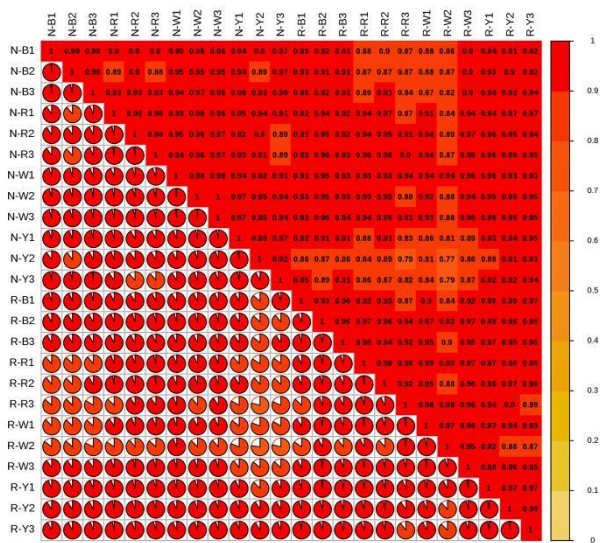

(B)

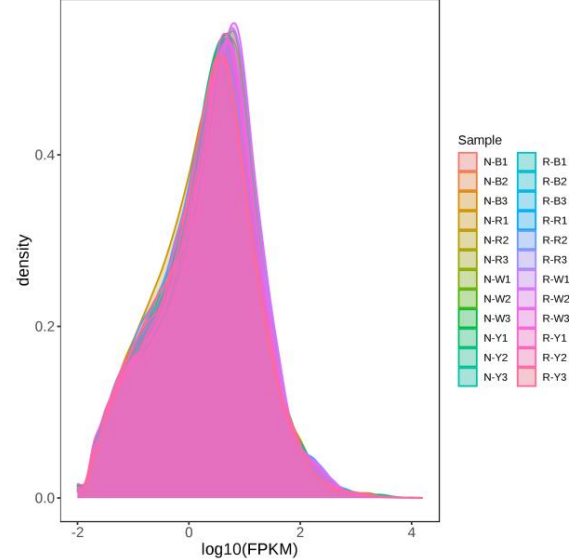

Supplementary Figure 6

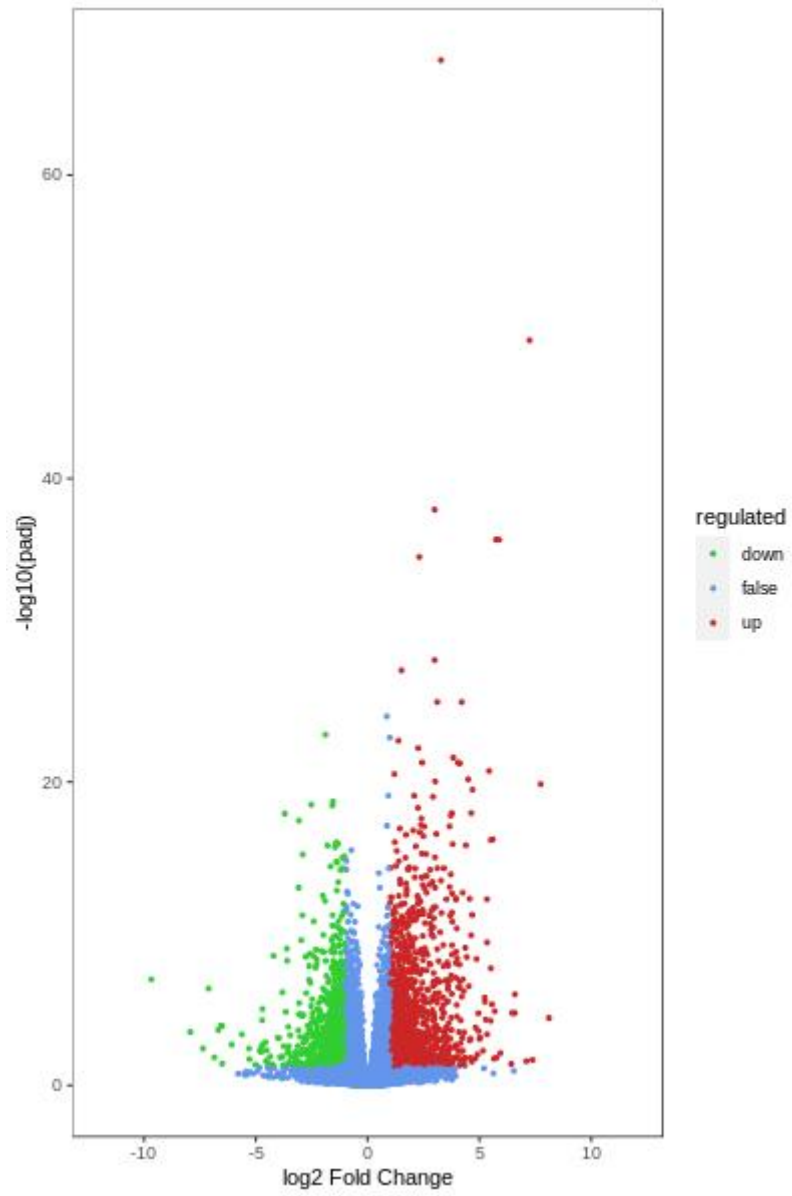

Supplement: Supplementary Figure 3 — Correlation diagram between samples. The abscissa represents the sample name, the ordinate represents the corresponding sample name, the color represents the correlation value, and the group is the group. [file Data_Sheet_8.PDF]

## Supplementary Figure 4

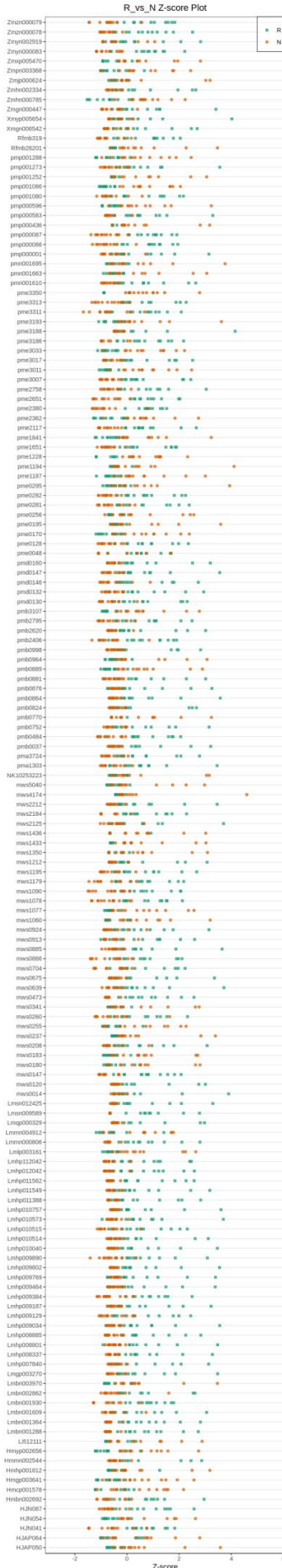

Supplement: Supplementary Figure 4 — Z-value diagram of differential metabolites. The horizontal coordinate is the value after normalization of the relative content of the substance, the vertical coordinate is the name of the metabolite, and the points of different colors represent different groups of samples. [file Data_Sheet_9.PDF]

Supplementary Figure 5

(A)

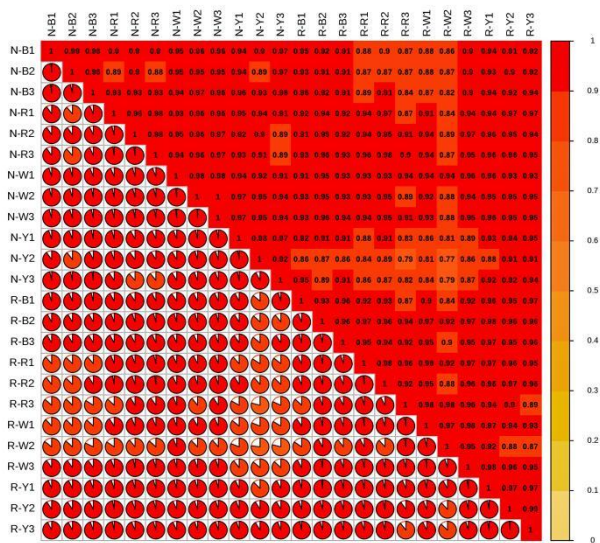

(B)

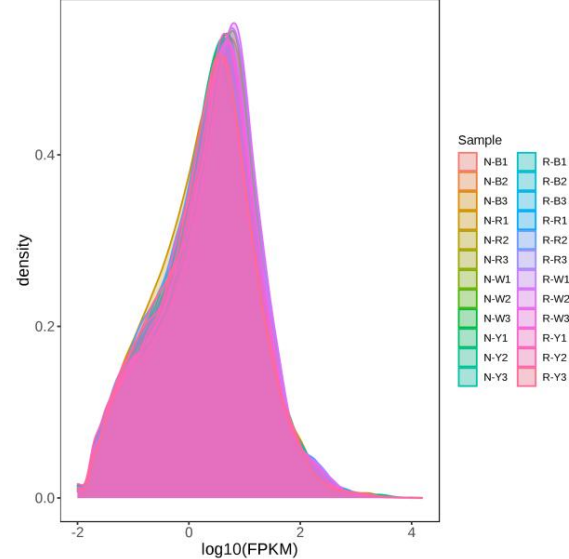

Supplement: Supplementary Figure 5 — (A) Correlation heat map. (B) Expression density distribution. The square of correlation heat map| r| between biological repeated samples must at least be greater than 0.8. The curves of different colors in the Expression density distribution represent different samples, and the abscissa represents the logarithm of the corresponding sample FPKM. [file Data_Sheet_10.PDF]

Supplementary Figure 6

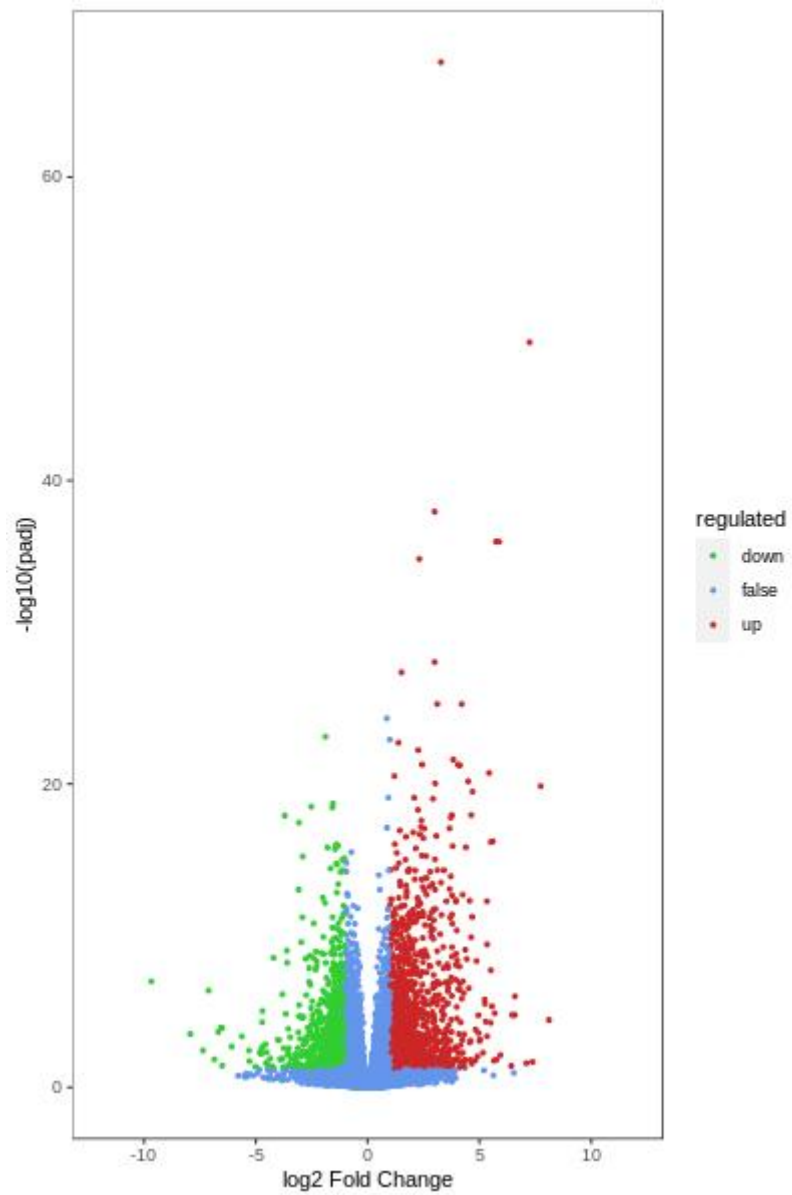

Supplement: Supplementary Figure 6 — Differential gene expression volcano plot. The horizontal coordinates indicate changes in gene expression level and the vertical coordinates indicate the level of significance of differentially expressed genes. Red dots represent upregulated differential genes, green dots represent downregulated differential genes and blue dots represent non-differentially expressed genes. [file Data_Sheet_11.PDF]

(A)

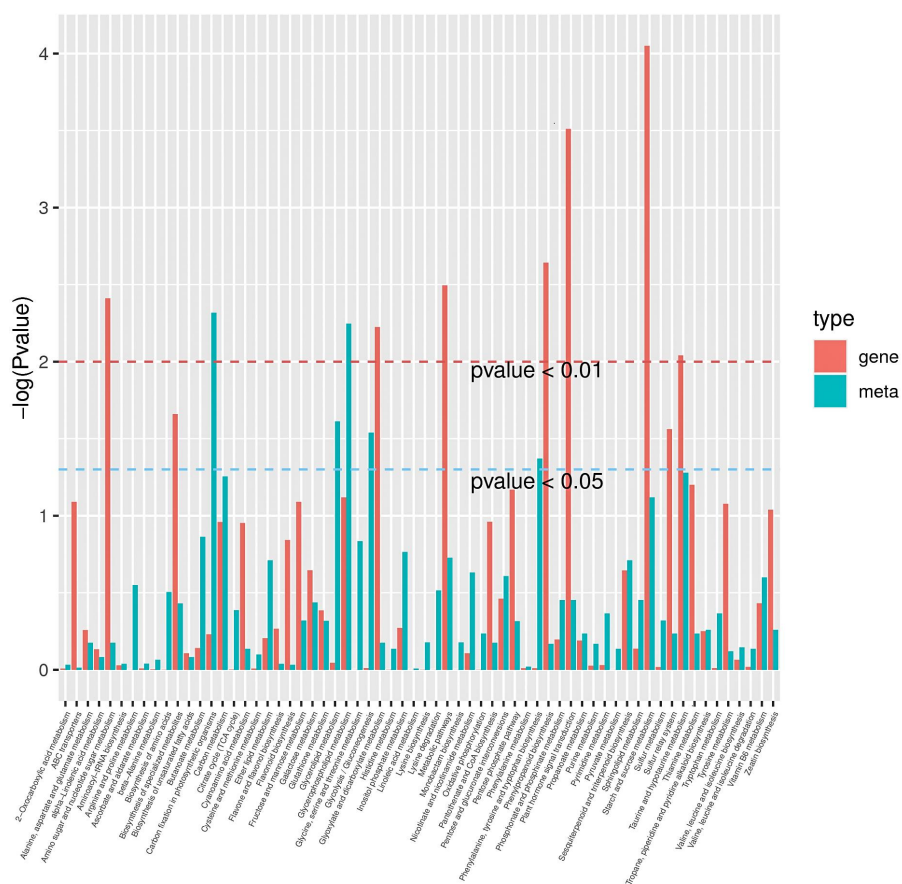

(B)

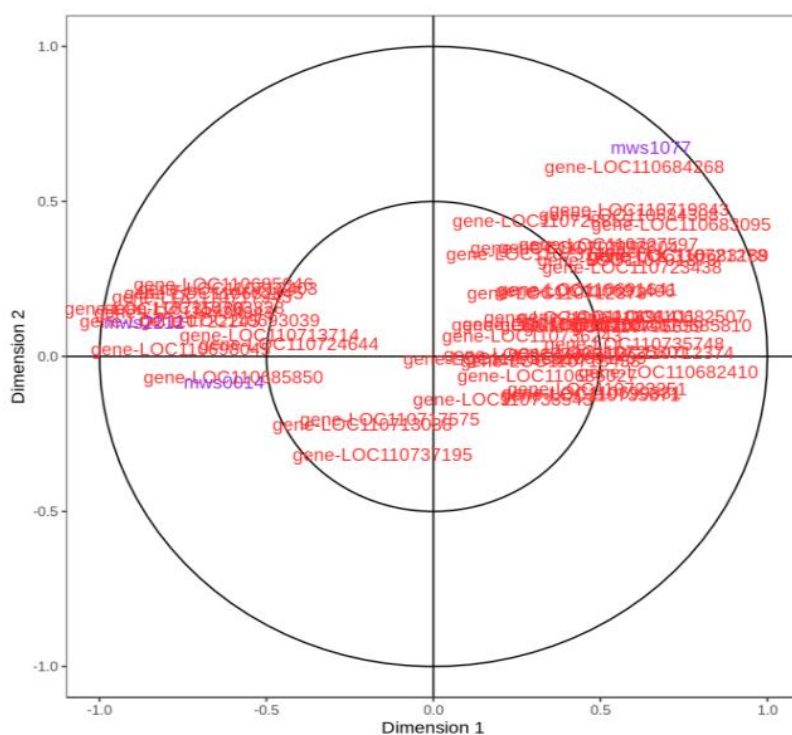

Supplement: Supplementary Figure 7 — (A) Kyoto Encyclopedia of Genes and Genomes (KEGG) enrichment analysis, p-Value histogram. (B) Phenylpropanoid biosynthesis canonical correlation analysis (CCA) plot. In the KEGG enrichment analysis, the p-value histogram shows the enrichment degree of pathways with both differential metabolites and genes. Phenylpropanoid biosynthesis canonical correlation analysis (CCA) plot is divided into four regions. In the same region, the farther away from the origin, the closer the data points are to each other, and the higher the correlation. [file Data_Sheet_12.PDF]
